# Supplementary material for: A route to metalloligands consolidated silver nanoclusters by grafting thiacalix[4]arene onto polyoxovanadates
Source: Nat Commun. 2023 Aug 31;14:5295. doi: 10.1038/s41467-023-41050-x (PMC10471715; doi:10.1038/s41467-023-41050-x)
Supplement: Supplementary file 1 — Supplementary information [file 41467_2023_41050_MOESM1_ESM.pdf]

Supplementary Information

A Route to Metalloligands Consolidated Silver Nanoclusters by Grafting Thiacalix[4]arene onto Polyoxovanadates

Zhi Wang,[1,2] Yan-Jie Zhu,[1,2] Bao-Liang Han,[1] Yi-Zhi Li,[1] Chen-Ho Tung,[1] and Di Sun[1*]

[1]School of Chemistry and Chemical Engineering, State Key Laboratory of Crystal Materials, Shandong University, Ji'nan, 250100, People's Republic of China.

[2]These authors contributed equally: Zhi Wang, Yan-Jie Zhu.

[*]To whom correspondence should be addressed. E-mail: dsun@sdu.edu.cn.

Section 1. Supplementary Methods

I. Materials and reagents

Solvents and reagents (Adamas-Beta®) were purchased from Shanghai Titan Scientific Co., Ltd. Unless otherwise noted, all of the chemicals were reagent grade and used without any further purification.

II. Infrared spectroscopy

The IR-ATR spectra were recorded on a Bruker Tensor II spectrometer in the frequency range of 4000-400 cm^{-1}.

III. Powder X-ray diffraction

Powder X-ray diffraction (PXRD) analyses were carried out on a microcrystalline powder using a Rigaku Oxford Diffraction XtaLAB Synergy-S diffractometer using Cu radiation (λ = 1.54184 Å). The PXRD patterns were processed with the *CrysAlis^{Pro}* software suite using the powder function.

IV. ^{1}H NMR analysis

^{1}H NMR spectrum was recorded in $CDCl_3$ at 298 K on Bruker AM-400 spectrometer.

V. Elemental analysis

The elemental analyses (C, H and N) were determined on a Vario EL Cube analyzer.

VI. Elemental mapping images

Morphology of the samples and elemental composition analyses were measured using an SU-8010 field emission scanning electron microscope (FESEM; Hitachi Ltd., Tokyo, Japan) equipped with an Oxford-Horiba Inca XMax50 energy dispersive X-ray spectroscopy (EDS) attachment (Oxford Instruments Analytical, High Wycombe, England).

VII. UV-Vis spectroscopy

UV-Vis spectra were recorded on a Thermo Scientific Evolution 220 UV-Vis spectrophotometer.

VIII. Mass spectra

Electrospray ionization mass spectra (ESI-MS) were recorded on a Bruker impact II high definition mass spectrometer, quadrupole and time-of-flight (Q/TOF) modules.

The data analysis of mass spectrum was performed based on the isotope distribution patterns using Compass Data Analysis software (Version 4.4).

IX. Photocurrent measurement

Photocurrent test and Mott-Schottky experiment were carried out on a CHI660E electrochemistry workstation. The crystals (0.7 μmol) of **Ag14** or **Ag43** and naphthol (0.5 wt. %, 15 μL) were dispersed in 0.5 mL EtOH and the mixture was sonicated for about 30 min. Then the solution was transferred by pipet dropped on the cleaned ITO glass and the coated film was obtained after evaporation under ambient atmosphere. The prepared ITO glass film was used as working electrode, a Pt wire as the counter electrode, and an Ag/AgCl electrode as the reference electrode in the aqueous solution of Na_2SO_4 (0.2 M) maintaining a bias voltage at 0.6 V.

X. Photothermal Conversion Studies

The crystals of **Ag14** (2 mg) were dispersed in 0.1 mL EtOH and sonicated for about 10 min, then evenly applied to the surface of the match head. Photothermal measurements were conducted using 660 nm laser (CNI Laser MDL-MD-660-1.3 W CE50050). The photothermal behavior of sample was monitored by thermal imaging camera (FLIR E54). Infrared photos and real-time temperatures were extracted from the video by FLIR tools software.

XI. X-ray Crystallography

Single crystals of **Ag2**, **Ag14** and **Ag43** with appropriate dimensions were chosen under an optical microscope and quickly coated with high vacuum grease (Dow Corning Corporation) to prevent decomposition. Single-crystal X-ray diffraction data of **Ag2**, **Ag14** and **Ag43** were collected on a Rigaku Oxford Diffraction XtaLAB Synergy diffractometer equipped with a HyPix-6000HE area detector at 100 K, 173 K and 100 K using Cu Kα (λ = 1.54184 Å) from Photon Jet micro-focus X-ray Source. The diffraction images were processed and scaled using the *CrysAlis^{Pro}* software suite.[1] The structures were solved using the charge-flipping algorithm, as implemented in the program *SUPERFLIP*[2] and refined by full-matrix least-squares techniques against F_o^2 using the SHELXL program[3] through the OLEX2 interface.[4] Hydrogen atoms at carbon were placed in calculated positions and refined isotropically by using a riding model.

Appropriate restraints or constraints were applied to the geometry and the atomic displacement parameters of the atoms in the cluster. All structures were examined using the Addsym subroutine of PLATON[5] to ensure that no additional symmetry could be applied to the models. Pertinent crystallographic data collection and refinement parameters are collated in Supplementary Table 1. Selected bond lengths and angles are collated in Supplementary Table 2.

Section 2. Supplementary Figures and Tables

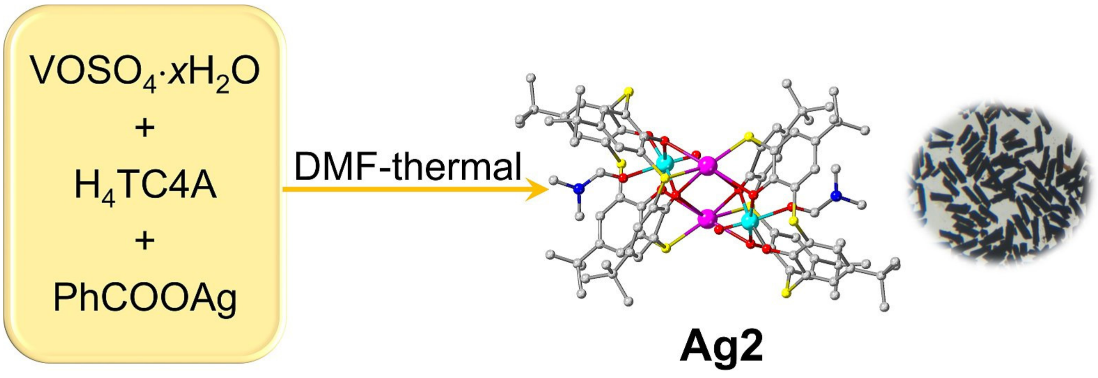

Supplementary Fig. 1: Synthetic route for Ag2. Color labels: purple, Ag; cyan, V; red, O; gray, C; yellow, S; blue, N.

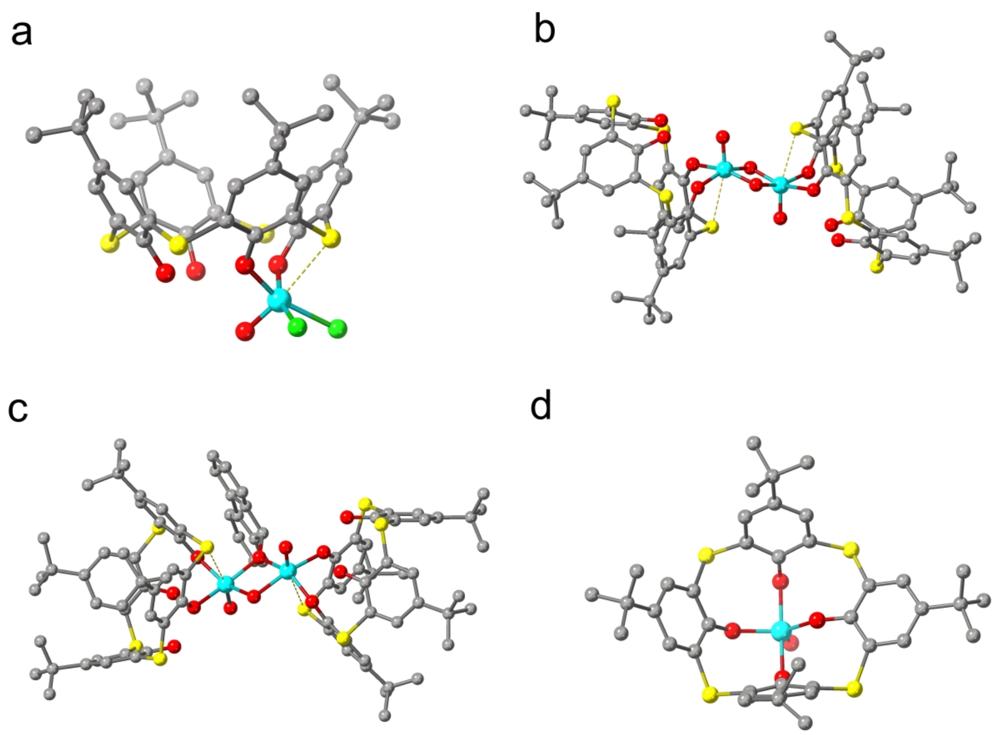

Supplementary Fig. 2: Previously reported oxovanadium(V) thiacalix[4]arene complexes $PPh_4[(H_2TC4A)VOCl_2]$ (a), $(PPh_4)_2[\{(H_2TC4AV(O)(\mu\text{-}O)\}_2]$ (b), $(PPh_4)_2[\{(H_2TC4A)V\}_2(\mu\text{-}OH)(\mu\text{-}OC_{13}H_9)]$ (c) and $PPh_4[(TC4A)V{=}O]$ (d) (H_4TC4A = *p*-tert-butylthiacalix[4]arene).[6] Color labels: cyan, V; gray, C; red, O; yellow, S; green, Cl.

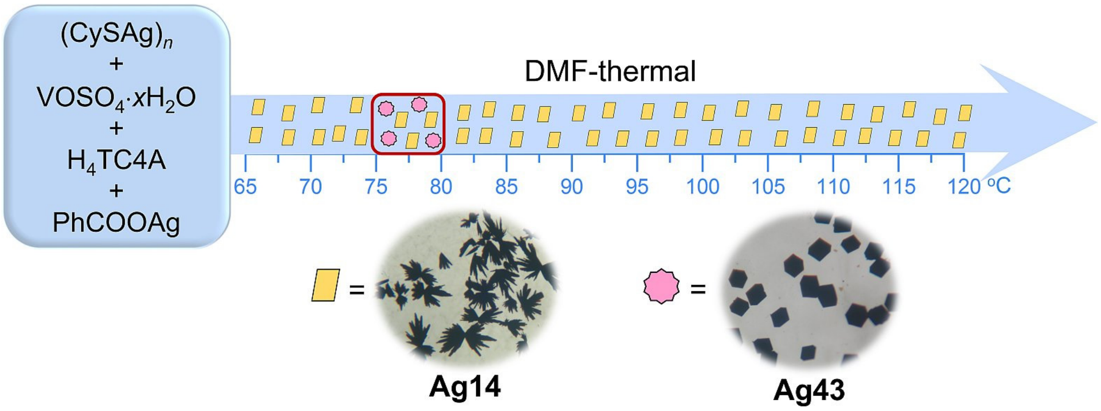

Supplementary Fig. 3: The temperature-dependent synthesis of Ag14 and Ag43.

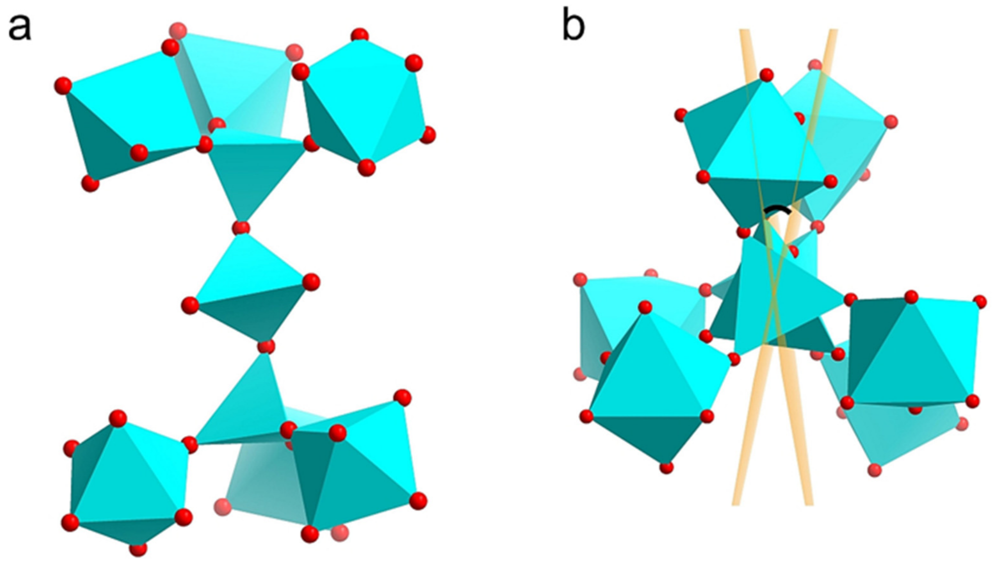

Supplementary Fig. 4: Side (a) and top (b) views of the polyoxovanadates (POVs). The dihedral angle of the orange planes to which the upper and lower {VO_6} belong is about 18.8°.

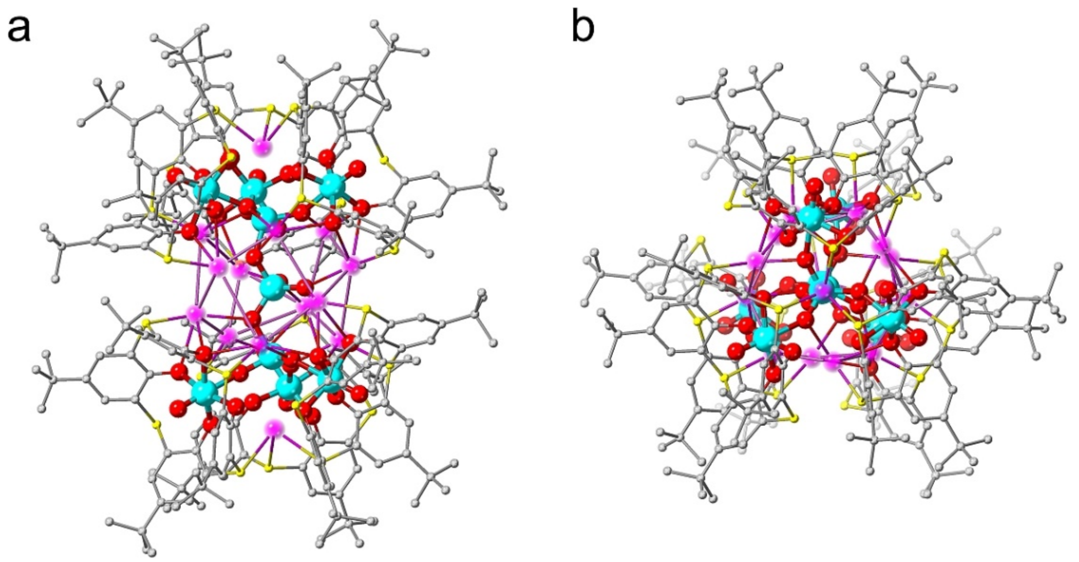

Supplementary Fig. 5: Side (a) and top (b) views of the 3D scaffold-like $[(TC4A)_6(V_9O_{16})]^{11-}$ metalloligand and Ag_{14} shell. Color labels: purple, Ag; cyan, V; red, O; gray, C; yellow, S.

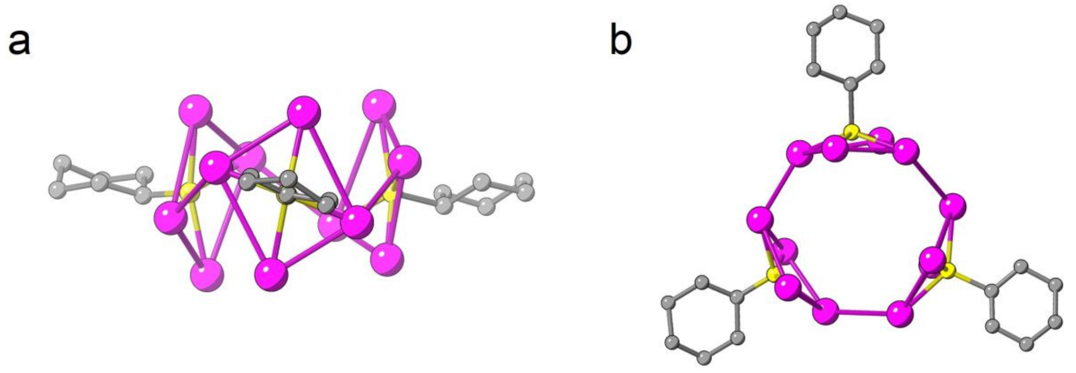

Supplementary Fig. 6: Side (a) and top (b) views of the μ_4 coordination mode of CyS$^-$ ligands towards silver shell in Ag14. Color labels: purple, Ag; gray, C; yellow, S.

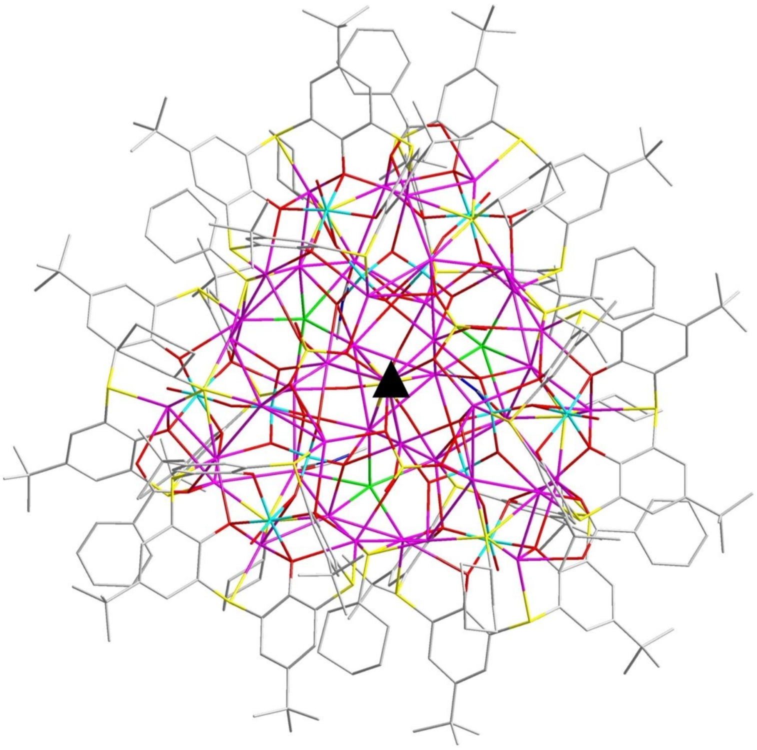

Supplementary Fig. 7: The crystallographic C_3 axis (▲) of Ag43. Color labels: purple, Ag; cyan, V; yellow, S; gray, C; red, O; green, Cl; blue, N.

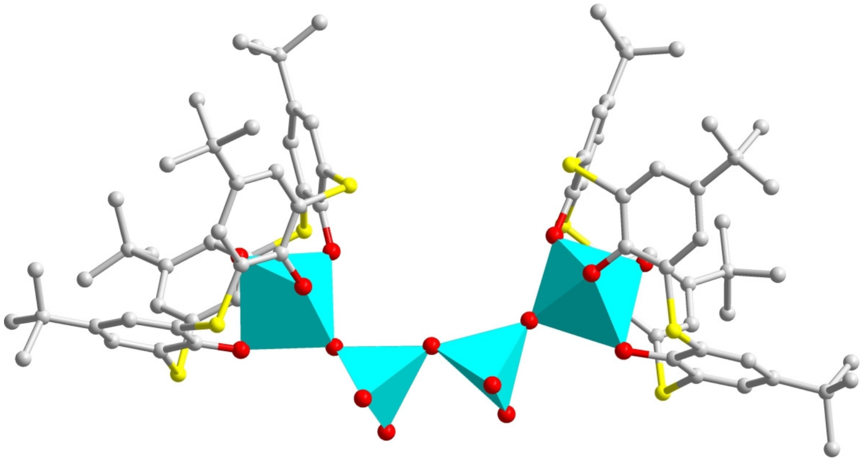

Supplementary Fig. 8: The structure of 1D arcuate $[(TC4A)_2(V_4O_9)]^{6-}$ metalloligand in Ag43. Color labels: red, O; gray, C; yellow, S; cyan polyhedron, POVs.

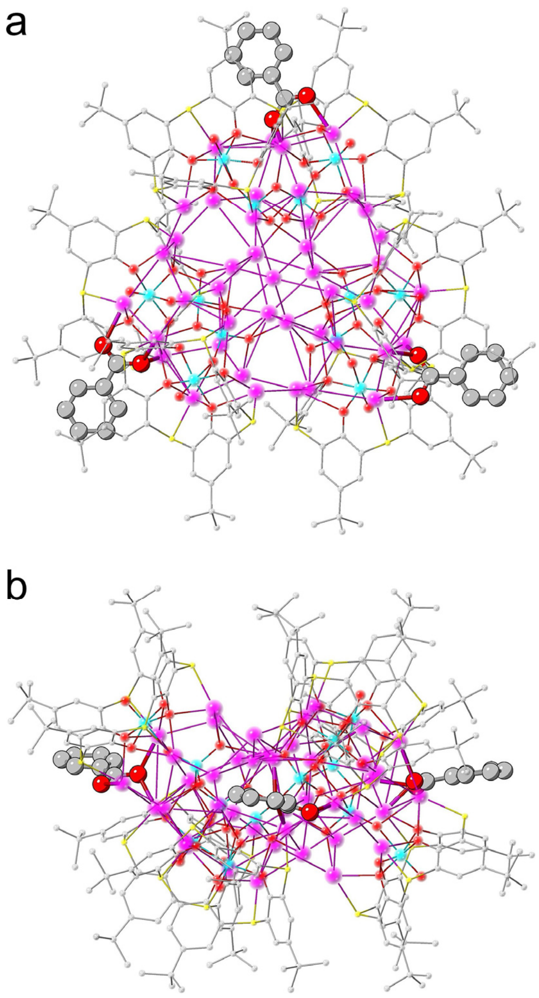

Supplementary Fig. 9: Top (a) and side (b) views of the coordination mode of PhCOO$^-$ ligands towards silver shell in Ag43. Color labels: purple, Ag; cyan, V; yellow, S; gray, C; red, O.

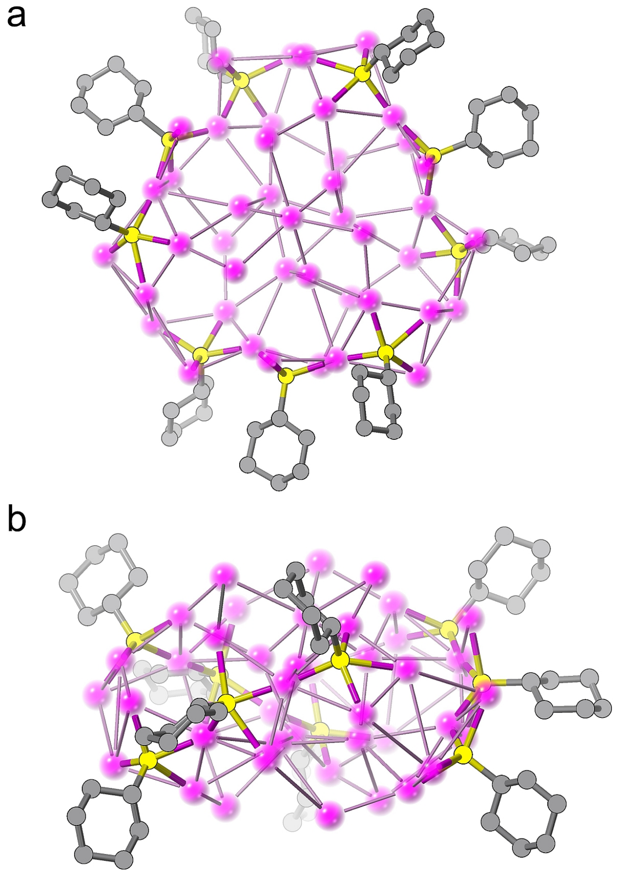

Supplementary Fig. 10: Top (a) and side (b) views of the μ_4 coordination mode of CyS$^-$ ligands towards silver shell in Ag43. Color labels: purple, Ag; gray, C; yellow, S.

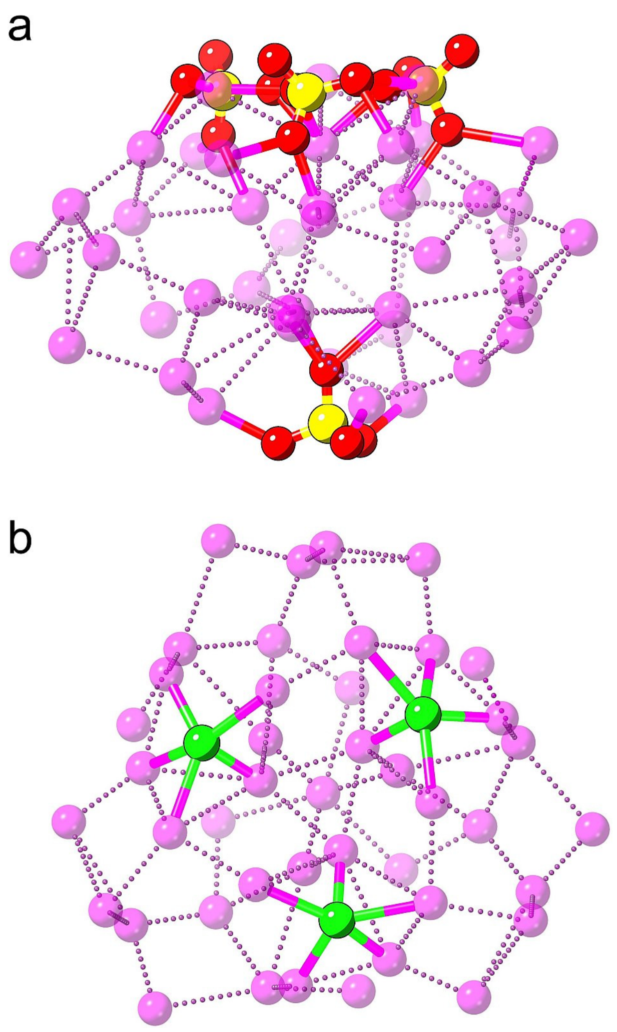

Supplementary Fig. 11: The coordination modes of SO_4^{2-} (a) and Cl^- (b) on the silver shell. Color labels: purple, Ag; yellow, S; red, O; green, Cl.

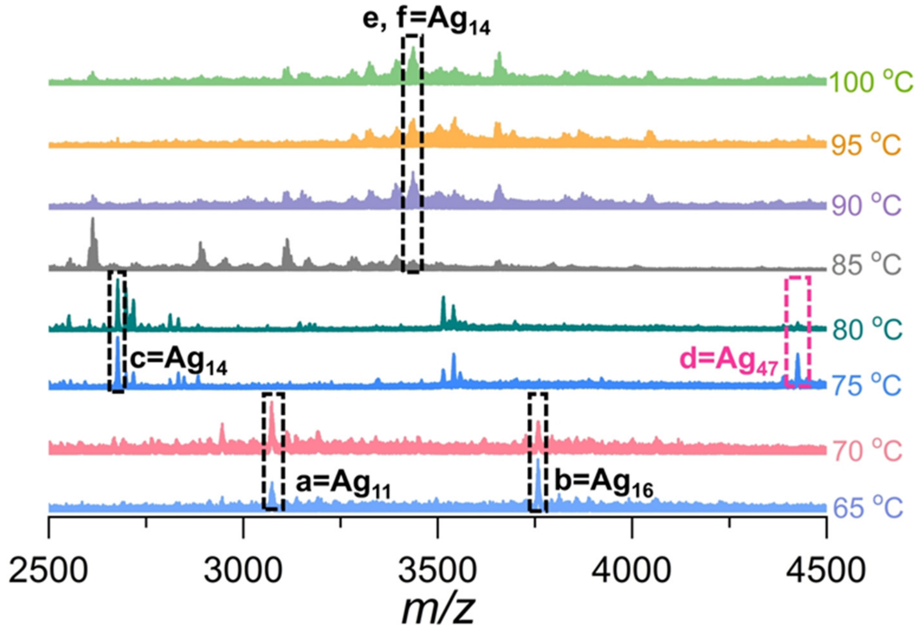

Supplementary Fig. 12: ESI-MS of the reaction solution at different temperatures.

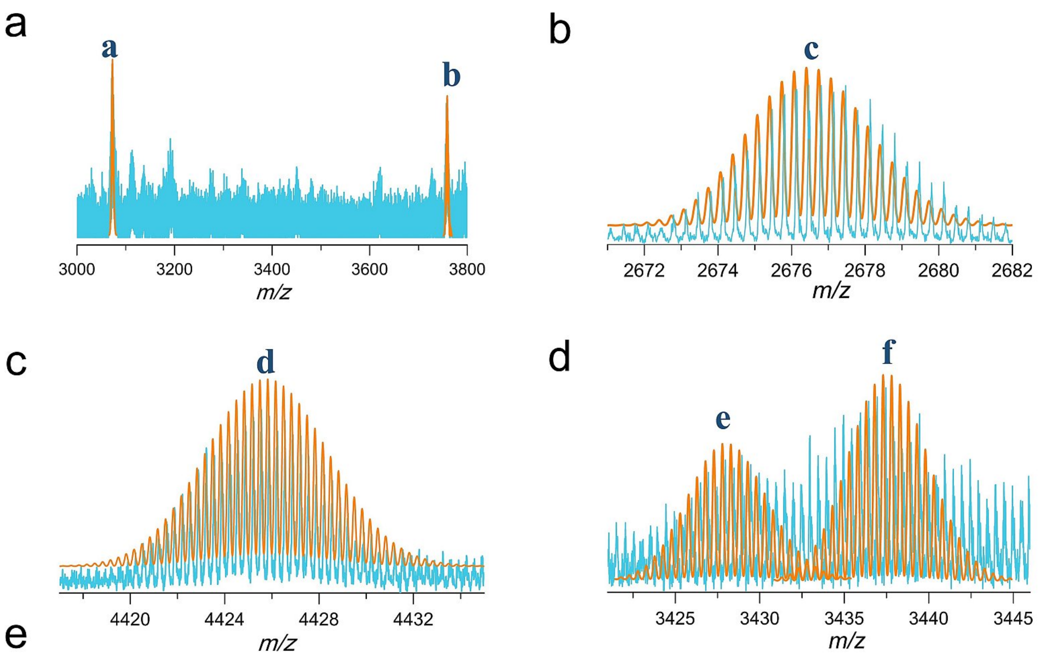

e

Species	Formulae	Exp. *m/z*	Sim. *m/z*
**a**	$\{Ag_{11}[(TC4A)_5(V_9O_{16})](CyS)_2(DMF)_5(CH_3OH)_2\}^{2+}$	3073.5032	3073.5043
**b**	$\{Ag_{16}[(TC4A)_6(V_9O_{16})](CyS)_3(DMF)_5(CH_3OH)_2\}^{2+}$	3758.9124	3758.8161
**c**	$\{Ag_{14}[(TC4A)_6(V_9O_{16})](DMF)_5(CH_3OH)_2(H_2O)\}^{3+}$	2676.4721	2676.3439
**d**	$\{Ag_{47}S[(TC4A)_2(V_4O_9)]_3(CyS)_9(PhCOO)_4Cl_3(SO_4)_4(DMF)_6(CH_3OH)_3(H_2O)\}^{3+}$	4425.5538	4425.5083
**e**	$\{Ag_{14}[(TC4A)_6(V_9O_{16})](CyS)(DMF)_2(CH_3OH)(H_2O)_2\}^{2+}$	3427.9278	3427.8057
**f**	$\{Ag_{14}[((TC4A)_6(V_9O_{16})](CyS)(DMF)_2(CH_3OH)(H_2O)_3\}^{2+}$	3437.4536	3437.3015

Supplementary Fig. 13: The experimental (blue trace) and simulated (orange trace) isotope patterns (a-d) and the assigned formulae (e) of a-f species.

a

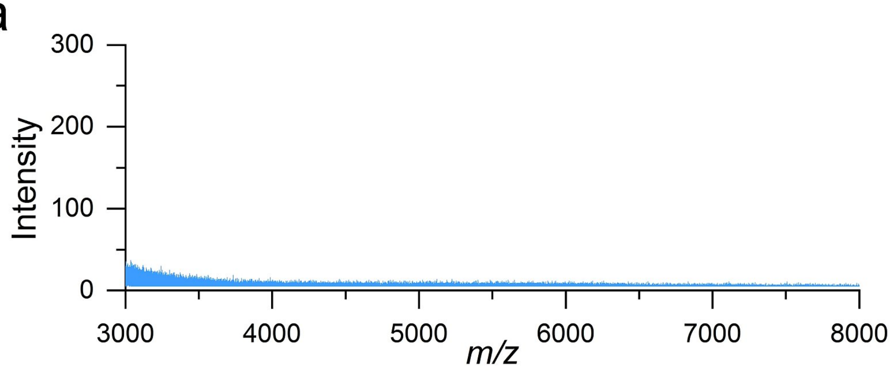

b

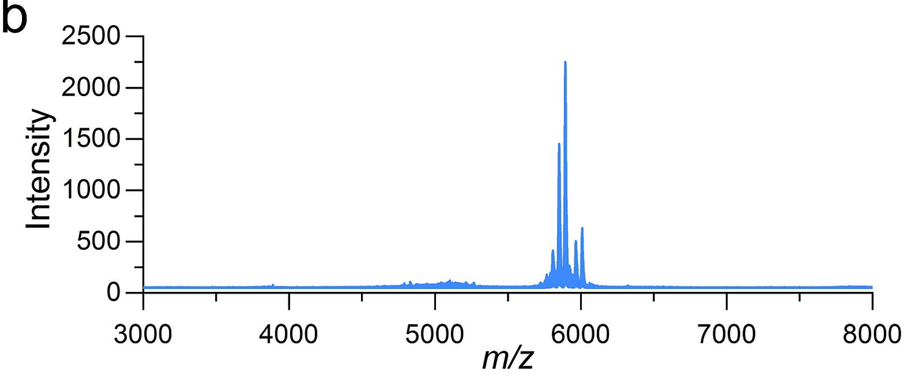

c

**Operating Parameters**	**Ag14**	**Ag43**
Temperature (^{o}C)	100	100
Capillary voltage (V)	5500	5500
Collision energy (V)	0	0
Quadrapole energy (V)	10	15
Nebulizer (Bar)	0.3	0.3
Nanobooster (Bar)	0.3	0.3
Sample injection rate (μL min^{-1})	20	20
Sample concentration (mol L^{-1})	5×10^{-5}	5×10^{-5}

Supplementary Fig. 14: Positive ion mode ESI-MS of Ag14 (a) and Ag43 (b) dissolved in CH_2Cl_2-CH_3OH mixed solvents under the same operating parameters (c).

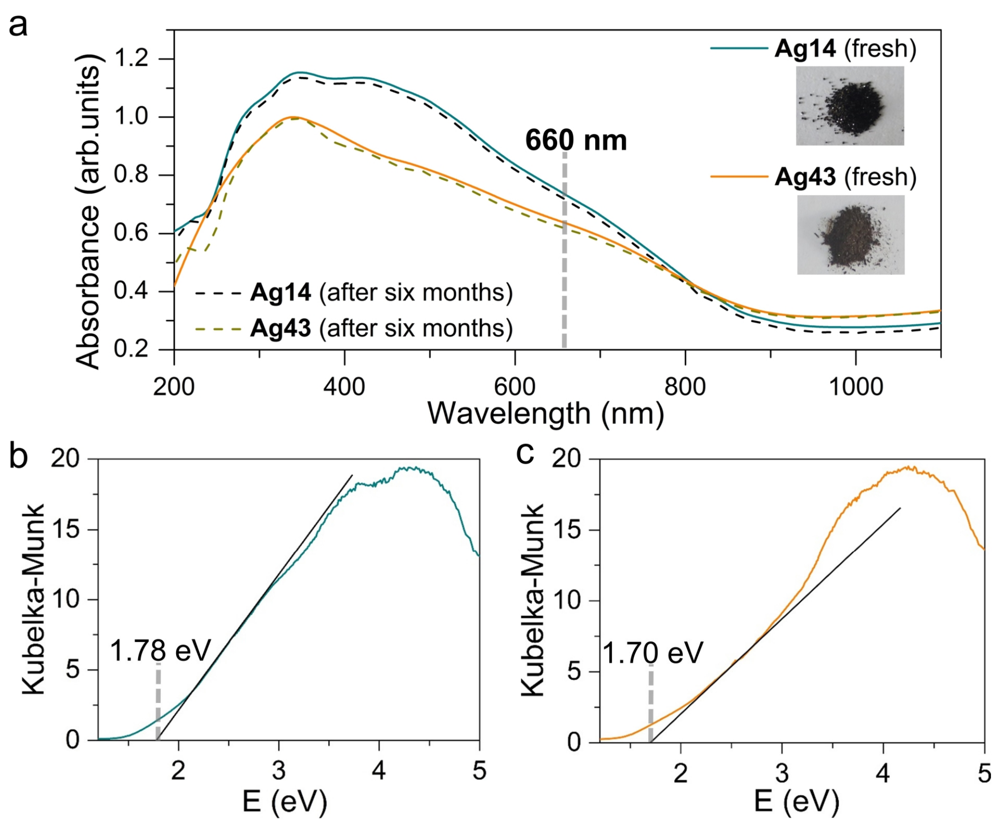

Supplementary Fig. 15: The UV-Vis spectra of fresh samples and samples stored in ambient conditions for six months (a), insets: photographs of Ag14 and Ag43. Diffuse reflectance UV-Vis spectra of Kubelka-Munk function *vs* energy and Tauc plots of of Ag14 (b) and Ag43 (c).

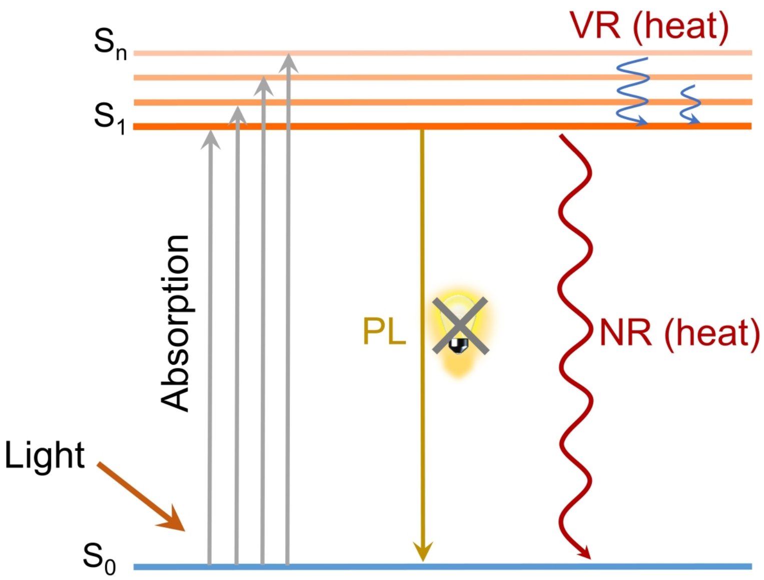

Supplementary Fig. 16. Simplified Jablonski diagram illustrating photophysical processes of Ag14 and Ag43 at room temperature. PL: photoluminescence, VR: vibrational relaxation, NR: non-radiative process.

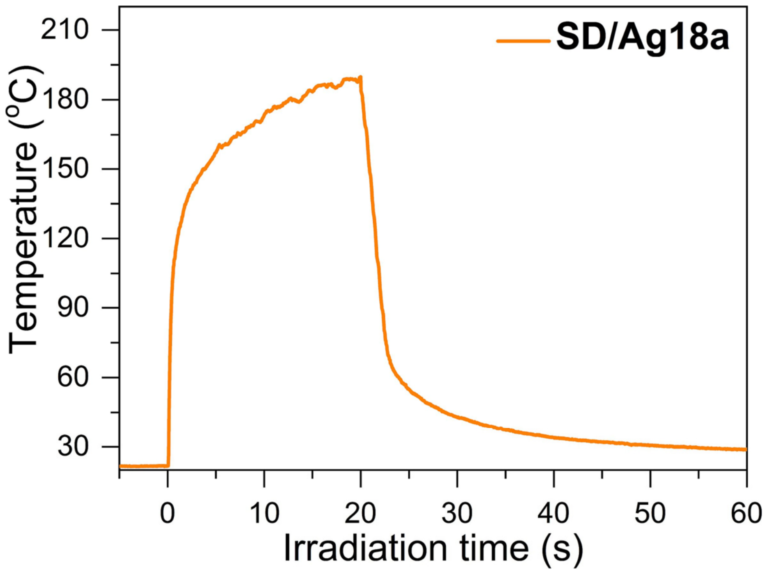

Supplementary Fig. 17: The plots of temperature evolution *vs* irradiation time for SD/Ag18a crystals[7] under 660 nm laser irradiation (0.9 W cm^{-2}) at a distance of 20 cm.

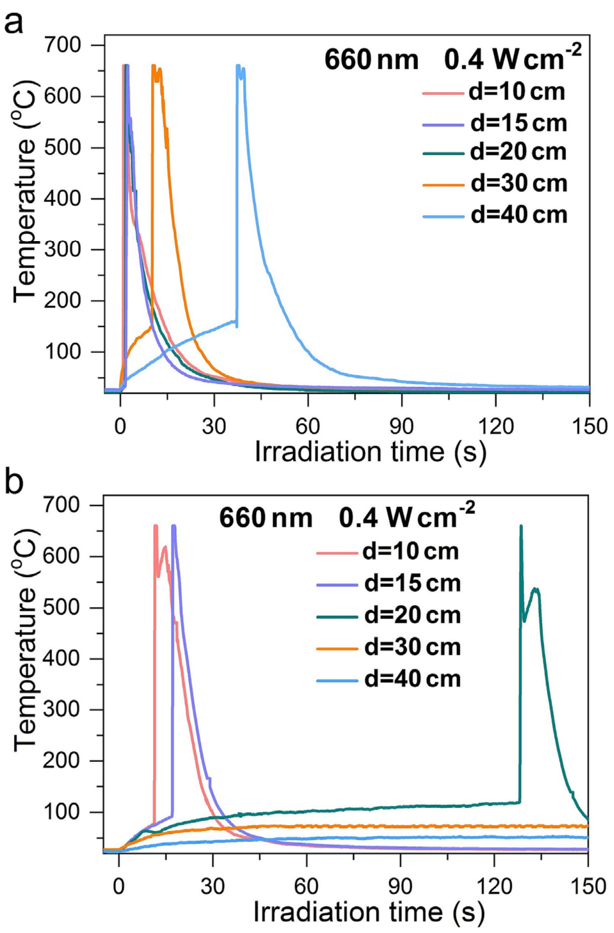

Supplementary Fig. 18: The plots of temperature evolution *vs* irradiation time for Ag14/match (a) and match (b) at different distances with the power of 0.4 W cm^{-2}.

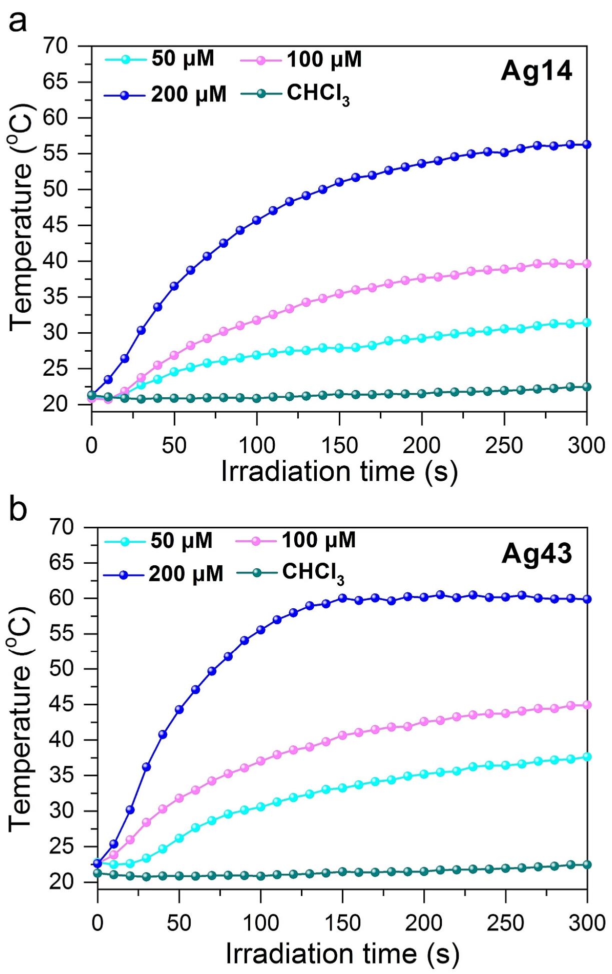

Supplementary Fig. 19: The plots of temperature evolution *vs* irradiation time for the $CHCl_3$ solutions of Ag14 (a) and Ag43 (b) at different concentrations under 660 nm laser irradiation (0.9 W cm^{-2}) at a distance of 15 cm.

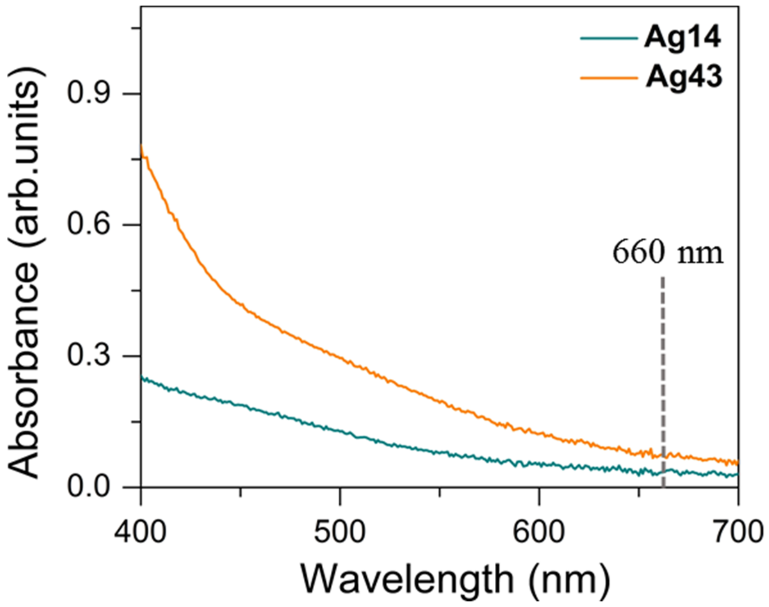

Supplementary Fig. 20: Compared absorbance in UV-Vis absorption spectra of the $CHCl_3$ solutions of Ag14 and Ag43 at 660 nm at the concentration of 20 μM.

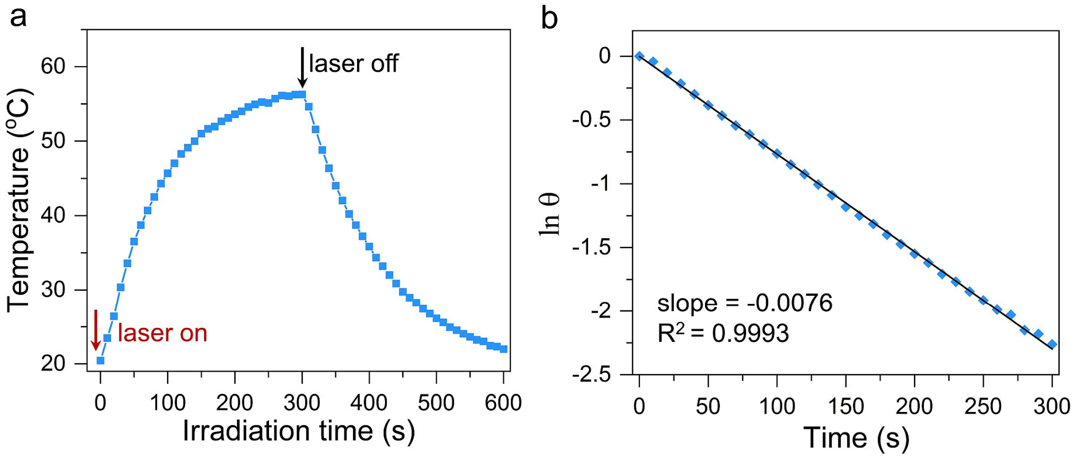

Based on the energy balance of the system, the photothermal conversion efficiency (η) can be calculated.[8-10]

$$\sum_i m_i C_{pi} \frac{dT}{dt} = Q_S - Q_{loss} \qquad (1)$$

where m (0.74 g) and C_p (1.189 J g^{-1} $^{o}C^{-1}$) are the mass and heat capacity of $CHCl_3$. Qs is the photothermal heat energy input by irradiating I, and Q_{loss} is thermal energy lost to the surroundings. When the temperature is maximum, the system is in balance.

$$Q_s = Q_{loss} = hs\Delta T_{max} \qquad (2)$$

where h is the heat transfer coefficient, s is the surface area of the container, ΔT_{max} is the maximum temperature change. The η is calculated from the following equations:

$$\eta = \frac{hs(\Delta T_{sample-}\Delta T_{solvent})}{I(1-10^{-A})} \qquad (3)$$

$$hs = \frac{\sum mC_p}{\tau_s} \qquad (4)$$

$$\tau_s = \frac{-t}{\ln\theta} \qquad (5)$$

$$\theta = \frac{(T_{amb} - T)}{(T_{amb} - T_{max})} \qquad (6)$$

where A is absorbance of sample at 660 nm, t is the time of the the cooling process, T_{amb} is 18 °C. According to the Equation (1-6), the η of the $CHCl_3$ solution of **Ag14** at a concentration of 200 μM under 660 nm laser irradiation was calculated. A fitting linear of lnθ-T with a slope of -0.0076, by which τ_s was calculated as 131.58 s (slope = -1 / τ_s). $\Sigma mC_p = \rho(CHCl_3)\cdot V(CHCl_3)\cdot C_p(CHCl_3)$ = 0.5 mL × 1.48 g mL^{-1} × 1.189 J g^{-1} $^{o}C^{-1}$ = 0.88 J $^{o}C^{-1}$. Therefore, hs = 0.88 / 131.58 = 6.69 × 10^{-3} J·$^{o}C^{-1}$·S^{-1}. ΔT_{sample} = 38.2 °C. $\Delta T_{solvent}$ = 1.2 °C. A_1 = 0.04 ×10 = 0.4 (Supplementary Fig. 20). Eventually, η_1 = 6.69 × 10^{-3} × (38.2 - 1.2) / [0.9 × (1 - $10^{-0.4}$)] = 45.69 %.

Supplementary Fig. 21: Heating and cooling curve of the $CHCl_3$ solution of Ag14 at a concentration of 200 μM under 660 nm laser irradiation (a). Fitting linear of ln*θ*-*T* (b).

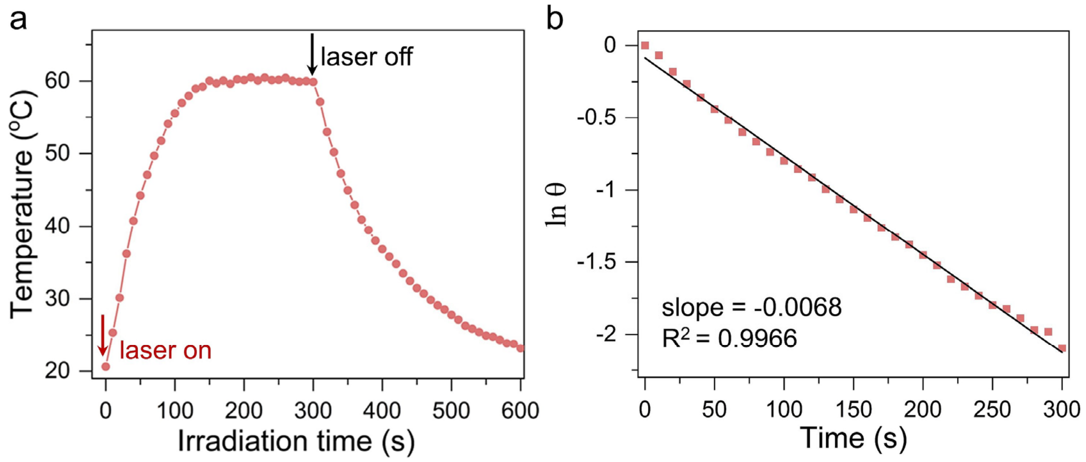

According to the Equation (1-6), the η of the $CHCl_3$ solution of **Ag43** at a concentration of 200 μM under 660 nm laser irradiation was calculated. A fitting linear of lnθ-T with a slope of -0.0068, by which τ_s was calculated as 147.06 s (slope = -1 / τ_s). $\Sigma mC_p = \rho(CHCl_3)\cdot V(CHCl_3)\cdot C_p(CHCl_3)$ = 0.5 mL × 1.48 g mL^{-1} × 1.189 J g^{-1} $°C^{-1}$ = 0.88 J $°C^{-1}$. Therefore, hs = 0.88 / 147.06 = 5.98 × 10^{-3} J·$°C^{-1}$·S^{-1}. ΔT_{sample} = 42 °C. $\Delta T_{solvent}$ = 1.2 °C. A_2 = 0.07 ×10 = 0.7 (Supplementary Fig. 20). Eventually, η_2 = 5.98 × 10^{-3} × (42 - 1.2) / [0.9 × (1 - $10^{-0.7}$)] = 33.87 %.

Supplementary Fig. 22: Heating and cooling curve of the $CHCl_3$ solution of Ag43 at a concentration of 200 μM under 660 nm laser irradiation (a). Fitting linear of lnθ-T (b).

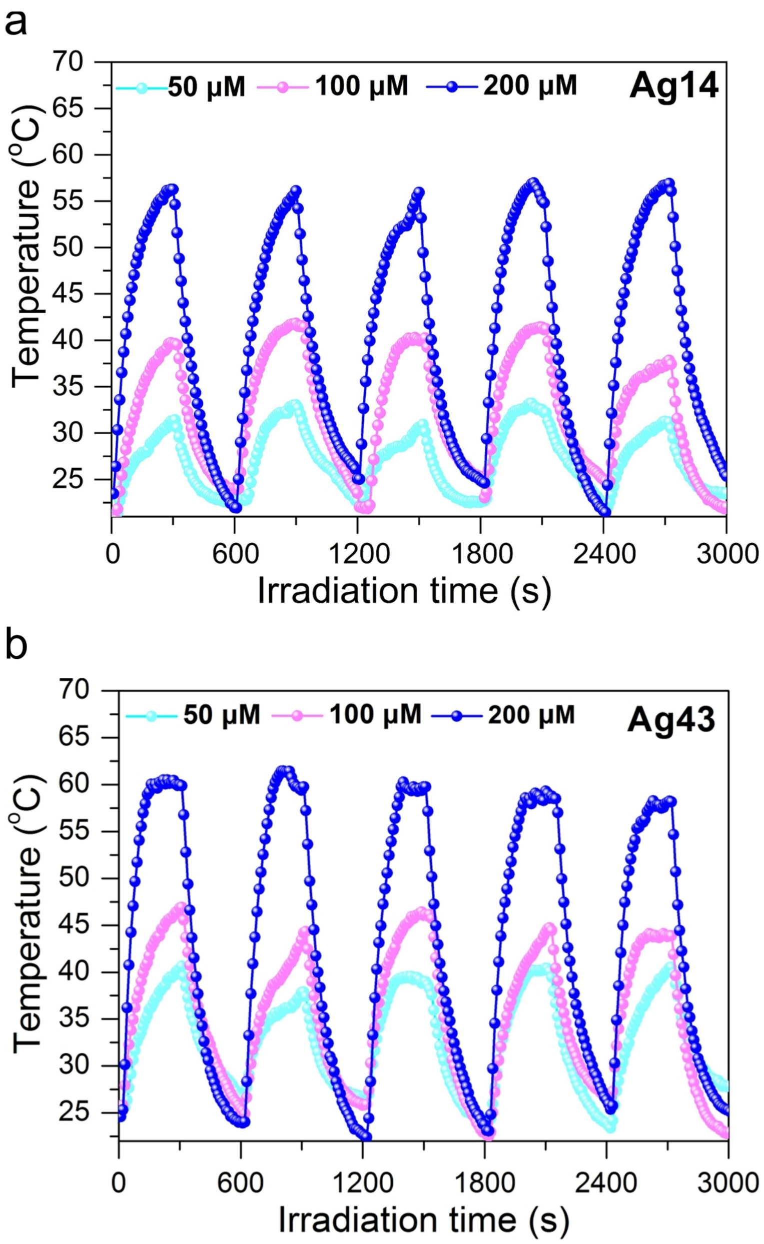

Supplementary Fig. 23: Photothermal heating and natural cooling cycles of the $CHCl_3$ solutions of Ag14 (a) and Ag43 (b) under 660 nm laser irradiation (0.9 W cm^{-2}) at a distance of 15 cm.

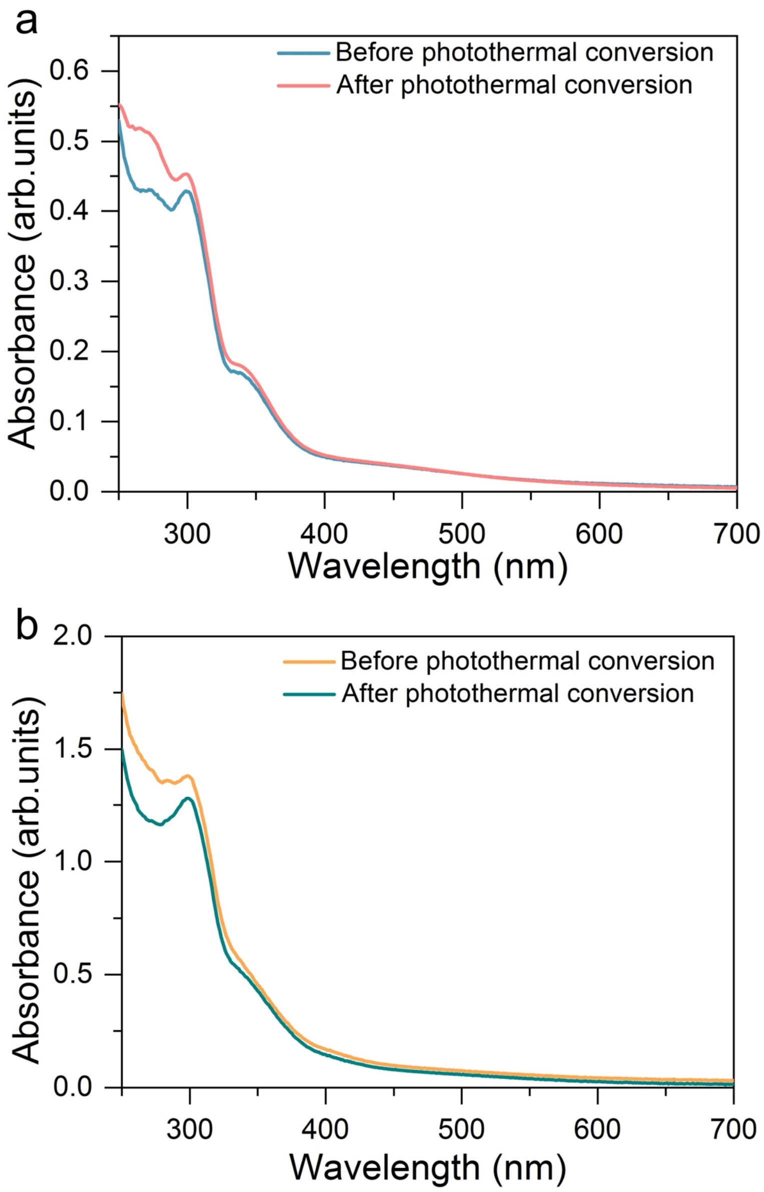

Supplementary Fig. 24: The compared UV-Vis absorption spectra of Ag14 (a) and Ag43 (b) before and after photothermal conversion.

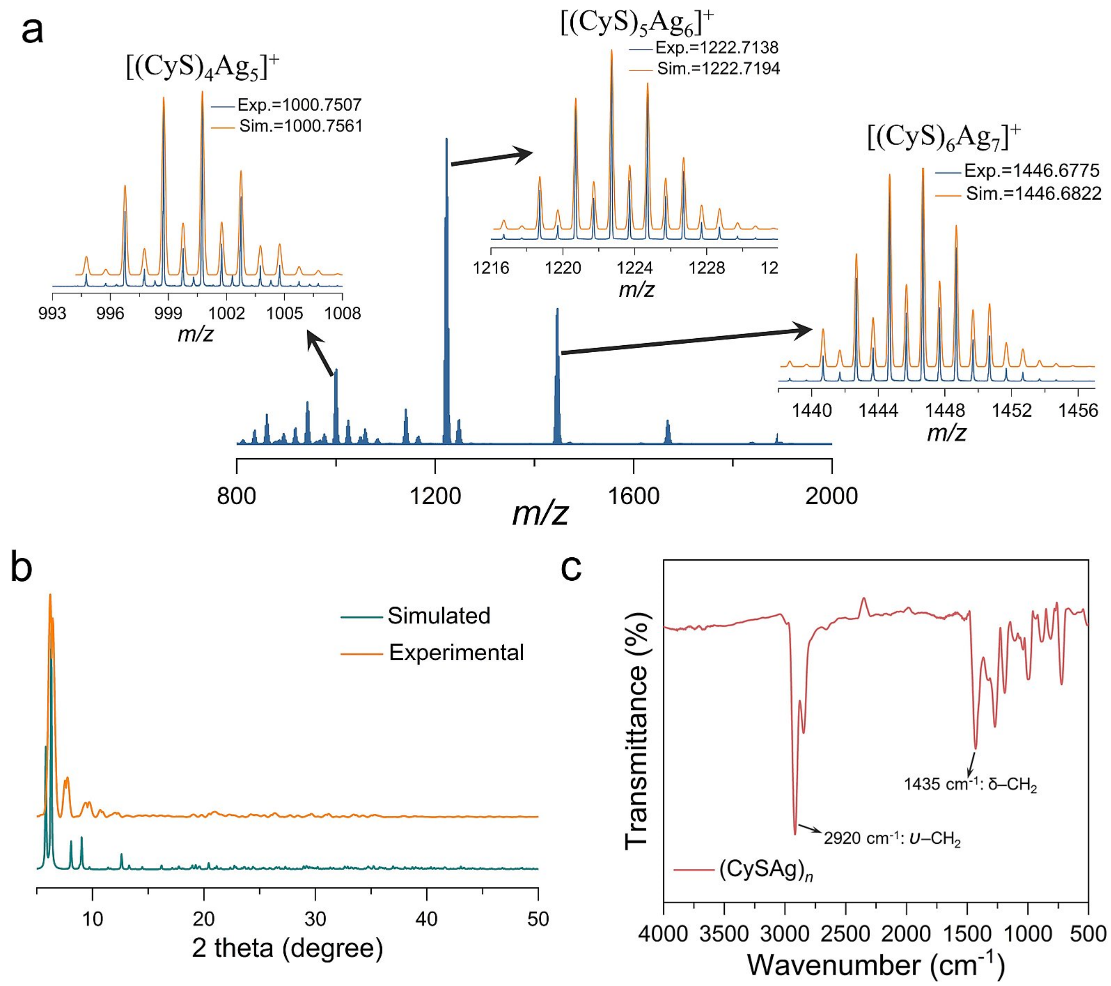

Supplementary Fig. 25: The ESI-MS (a), PXRD (b),[11] and IR spectra (c) of $(CySAg)_n$.

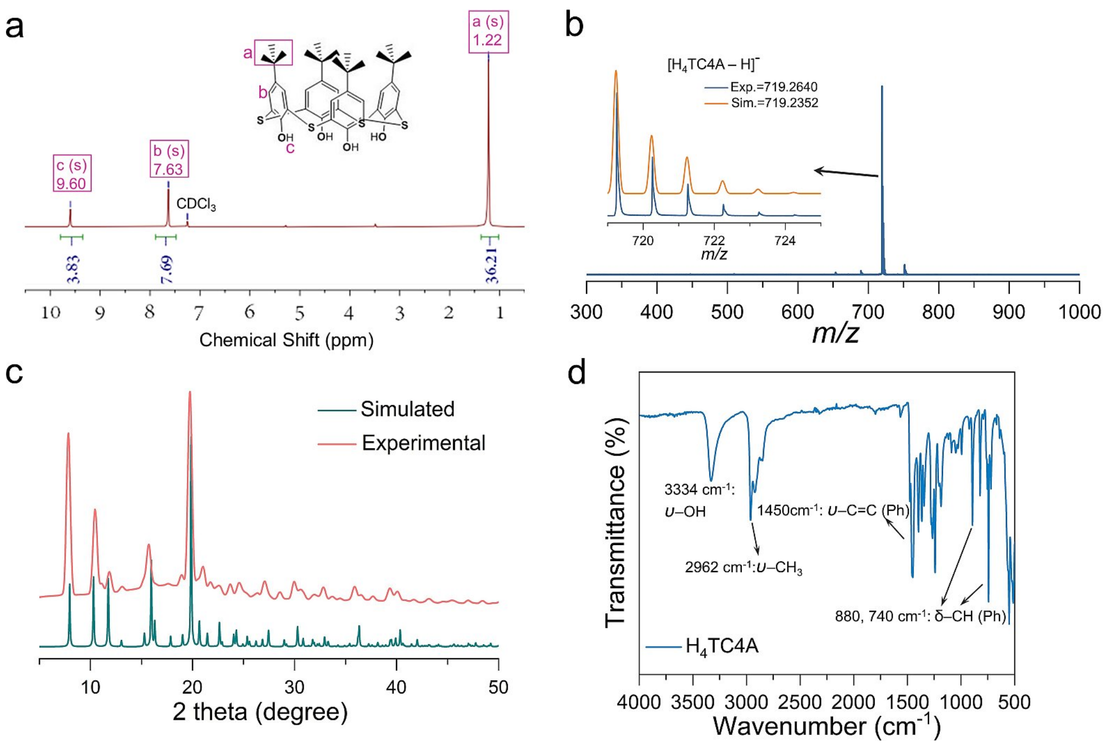

Supplementary Fig. 26: The ^{1}H NMR (a), ESI-MS (b), PXRD (c),[12] and IR spectra (d) of H_4TC4A.

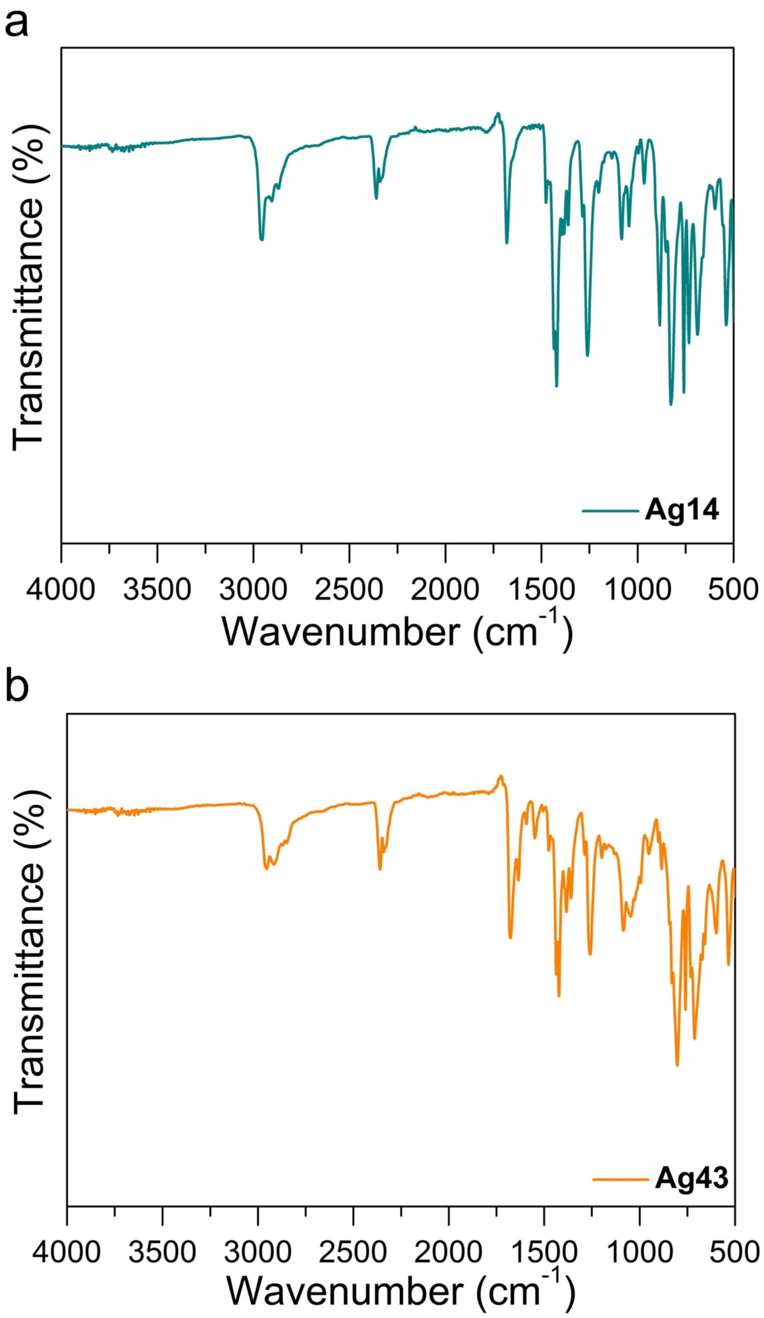

Supplementary Fig. 27: The IR spectra of Ag14 (a) and Ag43 (b).

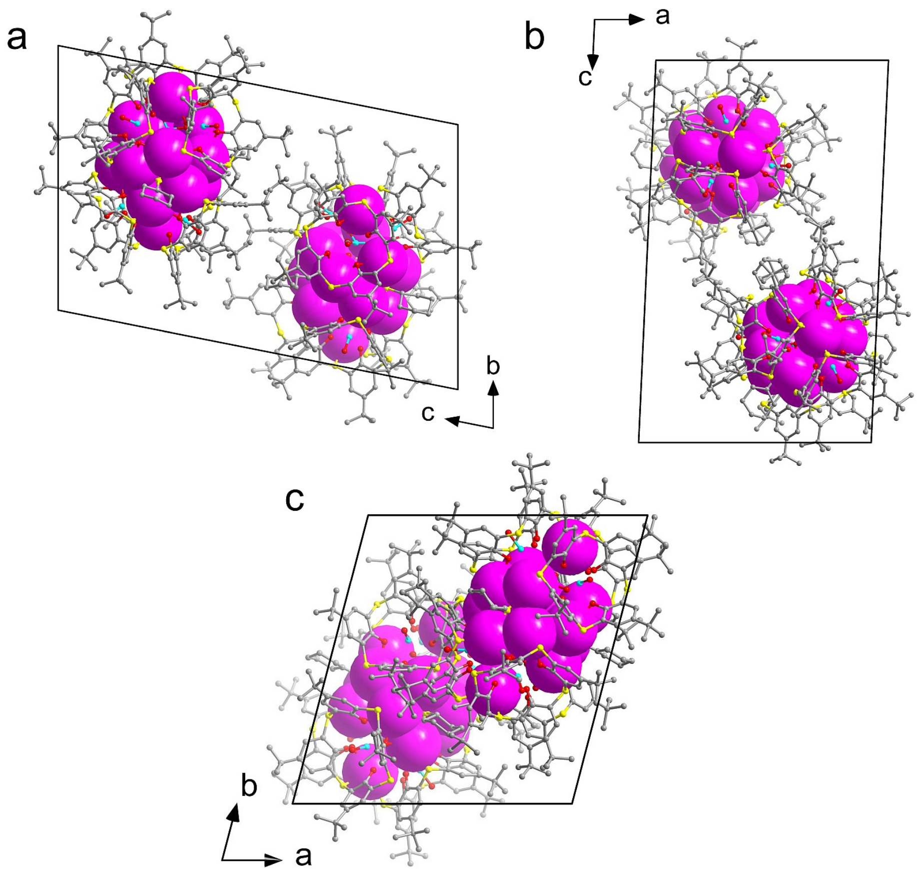

Supplementary Fig. 28: The molecule packing of Ag14 in a unit cell viewed along *a* (a), *b* (b), and *c* (c) axes. Color labels: purple, Ag; cyan, V; gray, C; yellow, S; red, O.

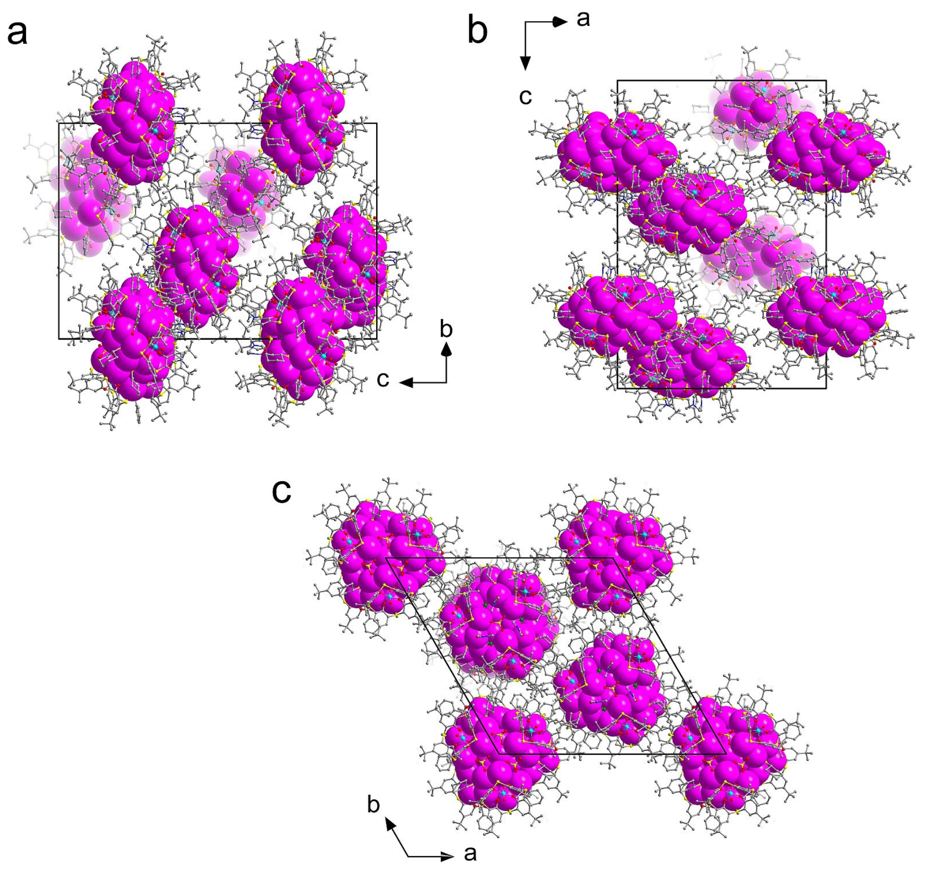

Supplementary Fig. 29: The molecule packing of Ag43 in a unit cell viewed along *a* (a), *b* (b), and *c* (c) axes. Color labels: purple, Ag; cyan, V; gray, C; yellow, S; blue, N; red, O; green. Cl.

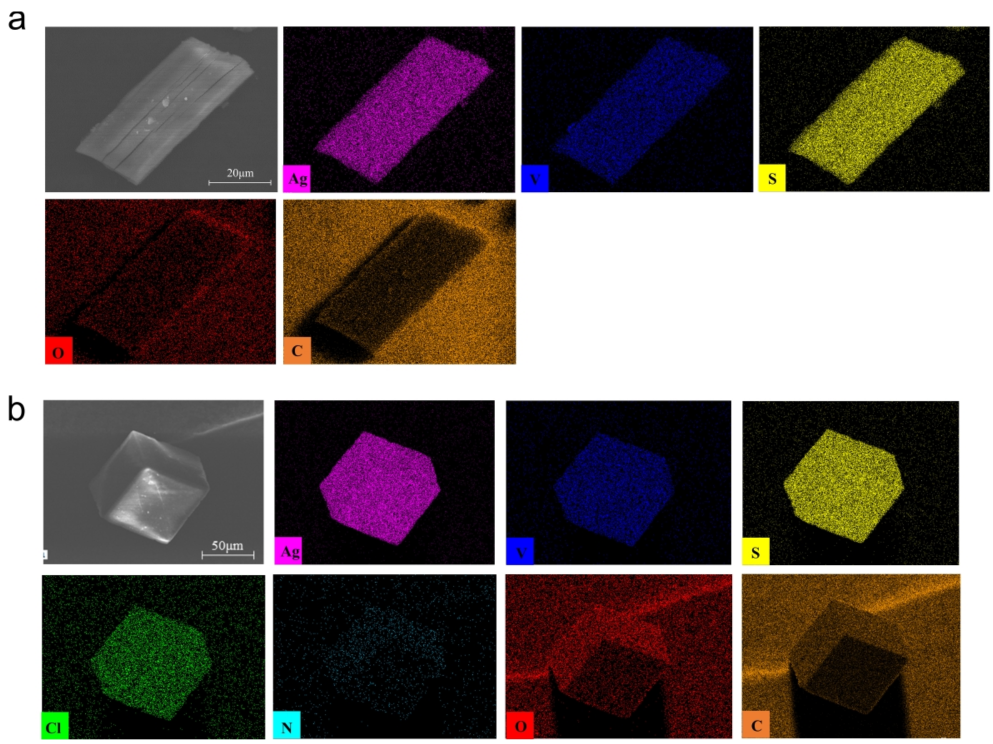

Supplementary Fig. 30: The morphology and elemental mapping of Ag14 (a) and Ag43 (b).

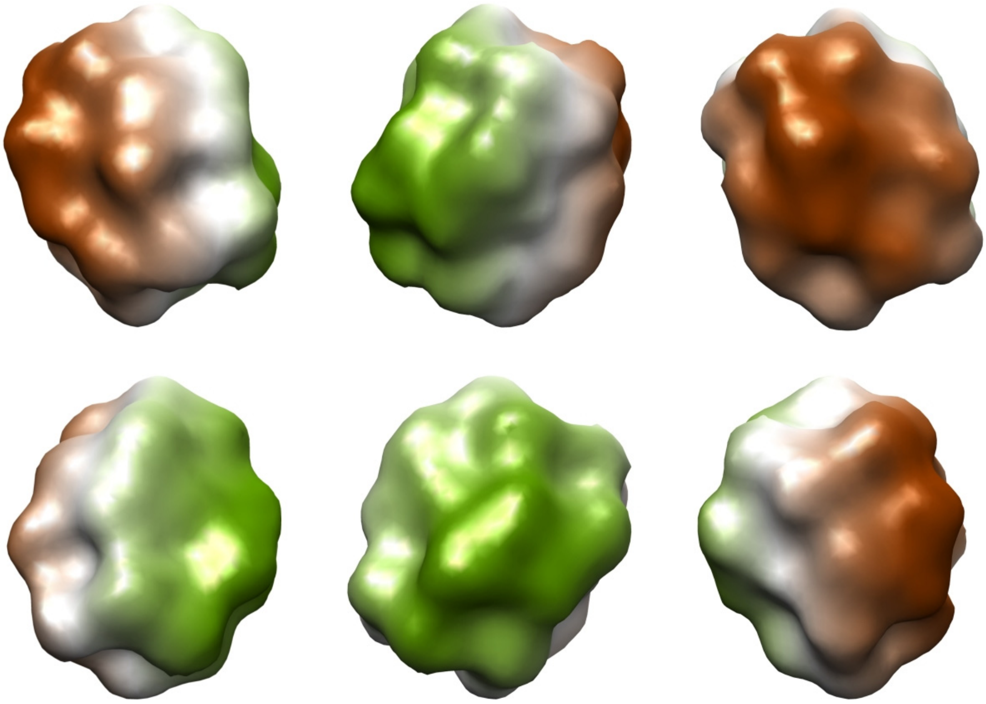

Supplementary Fig. 31: The surface of Ag14 calculated via 3V Volume Assessor program[13] by rolling a virtual probe (1.0 Å) on the surface viewed along six different orientations.

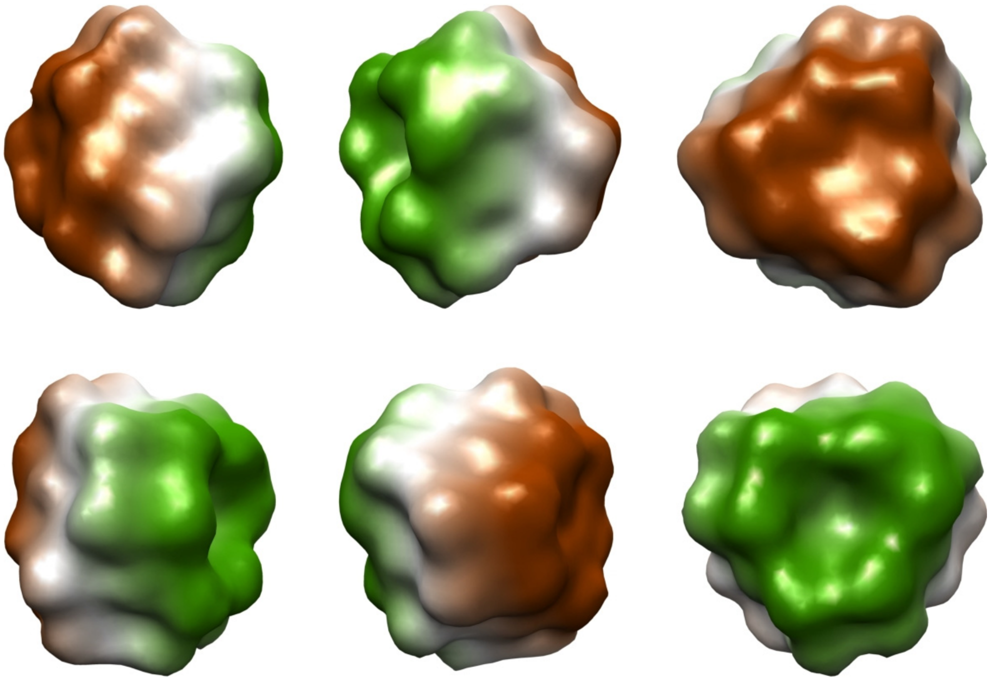

Supplementary Fig. 32: The surface of Ag43 calculated via 3V Volume Assessor program[13] by rolling a virtual probe (1.0 Å) on the surface viewed along six different orientations.

Supplementary Table 1: Crystal data collection and structure refinement for Ag2, Ag14, and Ag43.

Compound	**Ag2**	**Ag14**	**Ag43**
Empirical formula	$C_{104}H_{144}Ag_2N_8O_{18}S_8V_2$	$C_{258}H_{297}Ag_{14}O_{40}S_{27}V_9$	$C_{342}H_{441}Ag_{43}Cl_3N_9O_{82}S_{38}V_{12}$
Formula weight	2368.36	6872.20	12564.43
Temperature/K	100.00(10)	173.00(10)	100.00(10)
Crystal system	Monoclinic	Triclinic	Trigonal
Space group	*C2/c*	*P*-1	*R*-3
a/Å	26.7863(9)	21.6771(4)	39.2271(7)
b/Å	19.8358(5)	23.6072(5)	39.2271(7)
c/Å	25.9172(6)	35.1746(6)	50.0947(12)
α/°	90	79.0888(15)	90
β/°	113.379(3)	89.7613(13)	90
γ/°	90	75.5573(16)	120
Volume/$Å^3$	12640.0(7)	17098.6(5)	66756(3)
Z	4	2	6
$\rho_{calc}g/cm^3$	1.245	1.335	1.876
μ/mm^{-1}	5.363	10.188	19.153
F(000)	4944.0	6924.0	37014.0
Radiation	Cu Kα (λ = 1.54184 Å)	Cu Kα (λ = 1.54184 Å)	Cu Kα (λ = 1.54184 Å)
2Θ range for data collection/°	5.998 to 153.03	5.824 to 134.154	5.292 to 134.146
Reflections collected	40024	161788	103003
Independent reflections	12477 [R_{int} = 0.0561, R_{sigma} = 0.0529]	59209 [R_{int} = 0.0757, R_{sigma} = 0.0858]	26189 [R_{int} = 0.0760, R_{sigma} = 0.0471]
Data/parameters	12477/657	59209/3311	26189/1708
Goodness-of-fit on F^2	1.064	1.094	1.061
Final R indexes [I>=2σ (I)]	R_1 = 0.0855, wR_2 = 0.2515	R_1 = 0.1034, wR_2 = 0.3013	R_1 = 0.0775, wR_2 = 0.2155
Final R indexes [all data]	R_1 = 0.1015, wR_2 = 0.2681	R_1 = 0.1603, wR_2 = 0.3440	R_1 = 0.1069, wR_2 = 0.2459

Supplementary Table 2: Selected bond distances (Å) and angles (°) for Ag2, Ag14, and Ag43.

**Ag2**			
Ag1-S1	2.4820(16)	Ag1-S2[1]	2.4959(14)
S1-Ag1-S2[1]	150.92(6)		
Symmetry code: (1) 1-*x*,1-*y*,1-*z*.			
**Ag14**			
Ag1-Ag6	3.044(2)	Ag7-O6	2.471(10)
Ag1-V1	3.209(3)	Ag8-V1	3.238(2)
Ag1-S1	2.667(5)	Ag8-S5	2.748(6)
Ag1-S25	2.435(5)	Ag8-S13	2.713(5)
Ag1-O28	2.372(12)	Ag8-S20	2.685(4)
Ag2-Ag6	3.246(8)	Ag9-Ag13	3.1859(15)
Ag2-Ag5	3.175(6)	Ag9-S19	2.603(4)
Ag2-V2	3.173(5)	Ag9-S24	2.518(4)
Ag2-S1	2.290(6)	Ag9-O16	2.381(8)
Ag2-S21	2.762(6)	Ag10-Ag12	3.0370(15)
Ag2-O33	2.284(11)	Ag10-Ag5	3.3037(16)
Ag3-Ag14	3.215(2)	Ag10-S15	2.506(4)
Ag3-S23	2.580(5)	Ag10-S23	2.471(5)
Ag3-S27	2.669(4)	Ag10-O3	2.24(2)
Ag3-O21	2.410(7)	Ag10-O31	2.459(14)
Ag4-S2	2.839(6)	Ag11-Ag7	3.0711(15)
Ag4-S9	2.713(5)	Ag11-S7	2.475(4)
Ag4-S14	2.670(4)	Ag11-S19	2.477(4)
Ag5-S1	2.515(3)	Ag12-S18	2.511(3)
Ag5-S4	2.502(3)	Ag12-S23	2.601(4)
Ag5-O3	2.38(2)	Ag13-S12	2.475(3)
Ag6-Ag11	3.3535(17)	Ag13-S19	2.477(4)
Ag6-S1	2.440(3)	Ag13-O11	2.334(10)
Ag6-S22	2.520(3)	Ag14-S8	2.508(3)
Ag6-O18	2.550(8)	Ag14-S23	2.503(4)
Ag7-S6	2.510(3)	Ag14-O8	2.596(8)
Ag7-S19	2.603(4)	Ag14-O11	2.361(14)
S25-Ag1-S1	117.45(15)	S20-Ag8-S5	83.50(13)
O28-Ag1-S1	112.1(3)	S20-Ag8-S13	84.30(11)
O28-Ag1-S25	129.9(3)	S24-Ag9-S19	114.50(13)
S1-Ag2-S21	116.1(2)	O16-Ag9-S19	113.6(2)
O33-Ag2-S1	133.0(5)	O16-Ag9-S24	131.1(2)
O33-Ag2-S21	75.4(3)	S23-Ag10-S15	131.88(19)

S23-Ag3-S27	117.70(16)	O3-Ag10-S15	142.7(5)
O21-Ag3-S23	108.4(2)	O3-Ag10-S23	72.2(6)
O21-Ag3-S27	133.7(2)	O3-Ag10-O31	98.8(6)
S9-Ag4-S2	84.85(11)	O31-Ag10-S15	70.9(2)
S14-Ag4-S2	84.83(11)	O31-Ag10-S23	152.1(2)
S14-Ag4-S9	86.27(12)	S7-Ag11-S19	130.66(16)
S4-Ag5-S1	124.79(12)	S18-Ag12-S23	119.18(16)
O3-Ag5-S1	89.2(5)	S12-Ag13-S19	134.87(14)
O3-Ag5-S4	134.8(5)	O11-Ag13-S12	135.9(4)
S1-Ag6-S22	128.64(12)	O11-Ag13-S19	85.0(4)
S1-Ag6-O18	151.8(2)	S8-Ag14-O8	73.22(16)
S22-Ag6-O18	73.87(19)	S23-Ag14-S8	135.94(16)
S6-Ag7-S19	112.12(13)	S23-Ag14-O8	99.34(19)
O6-Ag7-S6	127.4(3)	O11-Ag14-S8	133.7(3)
O6-Ag7-S19	119.0(3)	O11-Ag14-S23	87.1(4)
S13-Ag8-S5	83.93(12)	O11-Ag14-O8	124.1(4)
**Ag43**			
Ag1-Ag4	3.218(16)	Ag7-O12	2.395(9)
Ag1-S2	2.563(19)	Ag8-Ag11	3.1784(14)
Ag1-S13[1]	2.312(14)	Ag8-Cl1[1]	2.873(3)
Ag1-O8[1]	2.51(2)	Ag8-S11	2.590(4)
Ag1-O10[1]	2.528(15)	Ag8-O2	2.293(8)
Ag2-Ag10	3.0252(13)	Ag8-O20	2.273(9)
Ag2-Ag13	2.9786(15)	Ag9-Cl1[1]	2.691(3)
Ag2-S4	2.660(3)	Ag9-S2	2.598(3)
Ag2-S5	2.670(3)	Ag9-S10	2.600(4)
Ag2-O4	2.600(7)	Ag10-S4	2.458(4)
Ag2-O11	2.529(7)	Ag10-S8	2.602(3)
Ag2-O12	2.331(9)	Ag10-O14	2.527(9)
Ag3-Ag6	3.0924(13)	Ag10-O22	2.445(9)
Ag3-Ag9[2]	3.0244(12)	Ag11-Cl1	2.874(3)
Ag3-Ag11	3.0683(14)	Ag11-S3	2.539(3)
Ag3-Cl1	2.837(3)	Ag11-O2	2.378(8)
Ag3-S2[2]	2.486(3)	Ag11-O18	2.326(11)
Ag3-S3	2.459(3)	Ag12-Ag13[1]	3.2029(15)
Ag3-O17	2.388(8)	Ag12-S12[1]	2.545(4)
Ag4-Ag9	3.0050(14)	Ag12-O1[1]	2.400(9)
Ag4-S2	2.376(3)	Ag12-O23[1]	2.543(11)
Ag4-S4	2.363(3)	Ag12-O24	2.307(13)
Ag4-O21	2.539(9)	Ag13-V3	3.170(2)
Ag5-Ag5[2]	3.2423(16)	Ag13-O1	2.345(7)
Ag5-Ag5[1]	3.2424(16)	Ag13-S4	2.427(3)
Ag5-Ag14[2]	3.2016(15)	Ag13-O23	2.261(12)

Ag5-Cl1	2.559(3)	Ag14-Ag15	2.9097(18)
Ag5-S1	2.677(4)	Ag14-S1	2.578(3)
Ag5-O2	2.540(7)	Ag14-O1	2.585(8)
Ag5-O17	2.521(8)	Ag14-O15	2.455(9)
Ag5-O19	2.480(13)	Ag14-O17^{1}	2.479(8)
Ag6-S3	2.445(3)	Ag14-O21	2.364(9)
Ag6-S6	2.645(3)	Ag15-S1	2.728(6)
Ag6-O4	2.405(8)	Ag15-O24^{2}	2.448(13)
Ag7-Ag11	3.2036(14)	Ag15-O24^{1}	2.448(13)
Ag7-S3	2.481(3)	Ag15-O24	2.448(13)
Ag7-S9	2.579(3)		
S13^{1}-Ag1-S2	124.1(7)	O2-Ag8-S11	144.8(2)
S13^{1}-Ag1-O8^{1}	77.5(5)	O20-Ag8-Cl1^{1}	92.4(3)
S13^{1}-Ag1-O10^{1}	78.3(4)	O20-Ag8-S11	90.5(3)
O8^{1}-Ag1-S2	147.1(8)	O20-Ag8-O2	107.5(3)
O8^{1}-Ag1-O10^{1}	66.2(5)	S2-Ag9-Cl1^{1}	109.61(9)
O10^{1}-Ag1-S2	92.3(6)	S2-Ag9-S10	107.92(10)
S4-Ag2-S5	92.19(10)	S4-Ag10-S8	137.63(12)
O4-Ag2-S4	153.3(2)	S4-Ag10-O14	146.18(18)
O4-Ag2-S5	69.44(18)	O14-Ag10-S8	72.35(18)
O11-Ag2-S4	90.50(19)	O22-Ag10-S4	112.9(3)
O11-Ag2-S5	163.07(18)	O22-Ag10-S8	83.6(2)
O11-Ag2-O4	101.8(2)	O22-Ag10-O14	80.2(3)
O12-Ag2-S4	124.8(2)	S3-Ag11-Cl1	102.95(9)
O12-Ag2-S5	96.2(2)	O2-Ag11-Cl1	90.73(18)
O12-Ag2-O4	77.8(3)	O2-Ag11-S3	117.6(2)
O12-Ag2-O11	96.2(3)	O18-Ag11-Cl1	87.2(3)
S2^{2}-Ag3-Cl1	108.41(9)	O18-Ag11-S3	124.8(3)
S3-Ag3-Cl1	106.14(10)	O18-Ag11-O2	116.4(3)
S3-Ag3-S2^{2}	135.35(10)	O1^{1}-Ag12-S12^{1}	145.9(2)
O17-Ag3-Cl1	87.4(2)	O1^{1}-Ag12-O23^{1}	77.4(3)
O17-Ag3-S2^{2}	90.8(2)	O24-Ag12-S12^{1}	123.8(4)
O17-Ag3-S3	118.29(19)	O24-Ag12-O1^{1}	90.3(4)
S2-Ag4-O21	93.15(19)	O24-Ag12-O23^{1}	111.8(4)
S4-Ag4-S2	169.39(11)	O1-Ag13-S4	139.1(2)
S4-Ag4-O21	97.4(2)	O23-Ag13-S4	131.4(3)
Cl1-Ag5-S1	161.06(8)	O23-Ag13-O1	84.3(4)
O2-Ag5-Cl1	94.8(2)	S1-Ag14-O1	91.95(18)
O2-Ag5-S1	102.87(19)	O15-Ag14-S1	145.4(3)
O17-Ag5-Cl1	91.04(19)	O15-Ag14-O1	87.5(3)
O17-Ag5-S1	84.1(2)	O15-Ag14-O17^{1}	93.4(3)
O17-Ag5-O2	84.0(2)	O17^{1}-Ag14-S1	87.09(18)
O19-Ag5-Cl1	101.1(3)	O17^{1}-Ag14-O1	179.0(3)

O19-Ag5-S1	86.6(3)	O21-Ag14-S1	111.7(2)
O19-Ag5-O2	87.44(18	O21-Ag14-O1	94.5(3)
O19-Ag5-O17	165.7(3)	O21-Ag14-O15	102.9(3)
S3-Ag6-S6	135.08(11)	O21-Ag14-O17^{1}	85.7(3)
O4-Ag6-S3	132.96(19)	O24^{1}-Ag15-S1	129.4(3)
O4-Ag6-S6	74.38(19)	O24^{2}-Ag15-S1	129.4(3)
S6-Ag7-S9	121.39(11)	O24-Ag15-S1	129.4(3)
O12-Ag7-S3	96.5(2)	O24^{1}-Ag15-O24	84.0(5)
O12-Ag7-S9	110.3(2)	O24^{2}-Ag15-O24	84.0(5)
S11-Ag8-Cl1^{1}	117.22(11)	O24^{1}-Ag15-O24^{2}	84.0(5)
O2-Ag8-Cl1^{1}	92.6(2)		
Symmetry codes: (1) *y*-*x*, 1-*x*, +*z*; (2) 1-*y*, 1+*x*-*y*, +*z*.			

Supplementary Table 3: The summary of metalloligand-protected silver nanoclusters (NCs).[7,14-17]

Metalloligand formula	Metalloligand structure	Silver NC formula	Silver NC structure	Ref.
TiL_3 (L= salicylate or 5-fluorosalicylate)	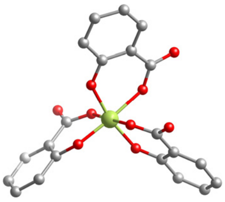	$Ti_4Ag_8(SA)_{12}$ (H_2SA = salicylicacid)	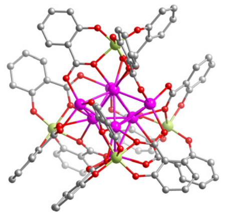	14
		$Ti_4Ag_{12}(S^iPr)_6(SA)_{10}(HSA)_2$	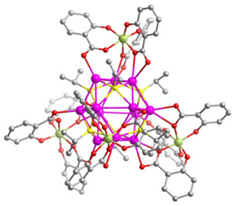	
		$Ti_4Ag_{22}(S^iPr)_{12}(SA)_{12}SO_4$	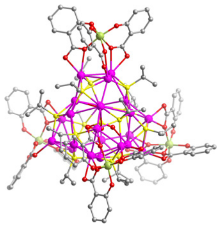	
		$Ti_4Ag_{42}(S)_4(S^iPr)_{18}(SA)_{12}(SO_4)_4$	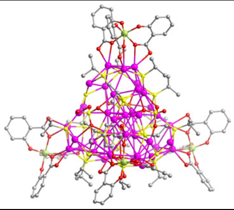	
	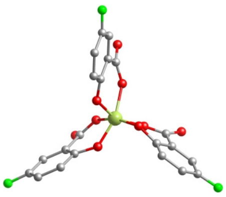	$Ti_4Ag_{36}(S^iPr)_{24}(SA\text{-}F)_{12}(SO_4)_2$ ($H_2SA\text{-}F$ = 5-fluorosalicylicacid)	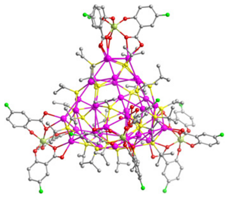	
$[Mo_2O_5(PTC4A)_2]^{6-}$ (H_4PTC4A = *p*-phenyl-thiacalix[4]arene)	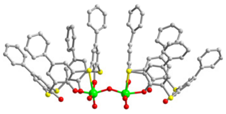	$[Ag_{18}(Mo_2O_5PTC4A)_6(EtS)_6(Tos)_2]\cdot 2Ag(CH_3CN)_3$	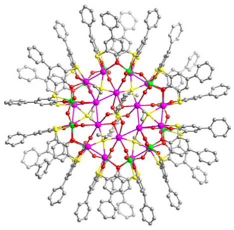	7

		$Ag_{18}S\{Mo_2O_5(PTC4A)_2[MoO_2(^nPrO)][(MoO(^nPrO)_2]\}_2(CyS)_6(Tos)_2(^nPrO)_2$	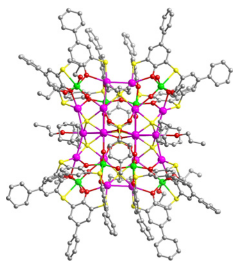	
MoO_3-TC4A (H_4TC4A = *p*-tert-butylthiacalix[4]arene)	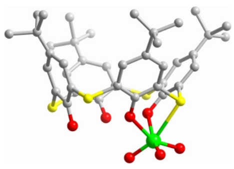	$NH_4[Cl@Ag_{42}(MoO_3\text{-}TC4A)_6(EtS)_{18}]$	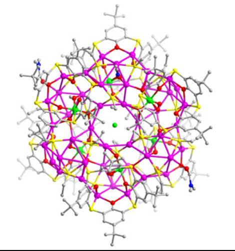	15
$Rh(aet)_3$ (Haet = 2-aminoethanethiolate)	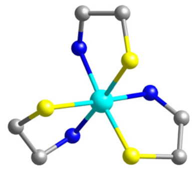	$[Ag_{46}S_{13}\{Rh(aet)_3\}_{14}]^{20+}$	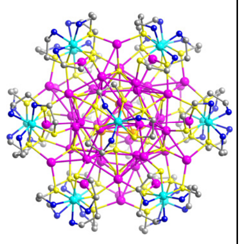	16
$Rh(apt)_3$ (Hapt = 3-aminopropanethiol)	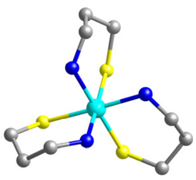	$[Ag_{11}S\{Rh(apt)_3\}_6]^{9+}$	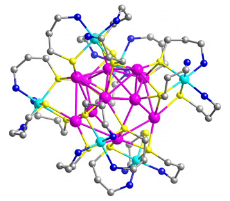	17
		$[Ag_{13}S\{Rh(apt)_3\}_6]^{11+}$	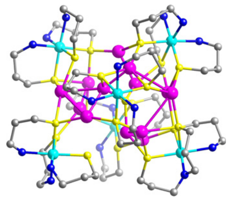	
$TC4A^{4-}$-POVs (H_4TC4A = *p*-tert-butylthiacalix[4]arene, POVs = polyoxovanadates)	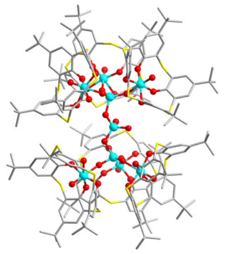	$Ag_{14}[(TC4A)_6(V_9O_{16})](CyS)_3$	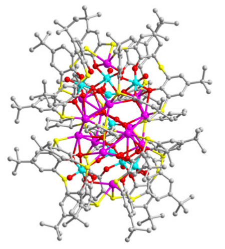	This work
	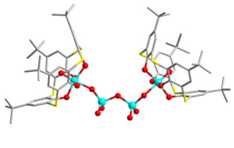	$Ag_{43}S[(TC4A)_2(V_4O_9)]_3(CyS)_9(PhCOO)_3Cl_3(SO_4)_4(DMF)_3\cdot 6DMF$	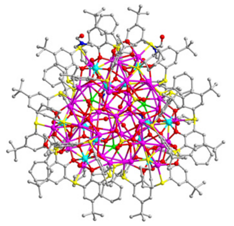	

Supplementary Table 4: The assigned formulae of the five species of Ag14 in CH_2Cl_2-CH_3OH mixed solvents identified by ESI-MS.

**Species**	**Formulae**	**Exp. *m/z***	**Sim. *m/z***
**1a**	$\{Ag_{14}[(TC4A)_6(V_9O_{16})](CyS)(DMF)(CH_3OH)_2\}^{2+}$	3398.2244	3398.2674
**1b**	$\{Ag_{14}[(TC4A)_6(V_9O_{16})](CyS)(CH_2Cl_2)_2\}^{2+}$	3405.7015	3405.7095
**1c**	$\{Ag_{14}[(TC4A)_6(V_9O_{16})](CyS)(CH_2Cl_2)_2(H_2O)\}^{2+}$	3414.6922	3414.6667
**1d**	$\{Ag_{15}[(TC4A)_6(V_9O_{16})](CyS)_2(CH_3OH)_2\}^{2+}$	3464.7599	3464.7175
**1e**	$\{Ag_{15}[((TC4A)_6(V_9O_{16})](CyS)_2(CH_3OH)_3\}^{2+}$	3480.7405	3480.7304

Supplementary Table 5: The assigned formulae of the four species of Ag43 in CH_2Cl_2-CH_3OH mixed solvents identified by ESI-MS.

**Species**	**Formulae**	**Exp. *m/z***	**Sim. *m/z***
**2a**	$\{Ag_{43}S[(TC4A)_2(V_4O_9)]_3(CyS)_9(PhCOO)Cl_3(SO_4)_4(H_2O)_2\}^{2+}$	5850.1935	5850.3182
**2b**	$\{Ag_{43}S[(TC4A)_2(V_4O_9)]_3(CyS)_9(PhCOO)Cl_3(SO_4)_4(CH_2Cl_2)(H_2O)_2\}^{2+}$	5892.7112	5892.7942
**2c**	$\{Ag_{44}S[(TC4A)_2(V_4O_9)]_3(CyS)_9(PhCOO)_2Cl_3(SO_4)_4(H_2O)_2\}^{2+}$	5964.6603	5964.7853
**2d**	$\{Ag_{44}S[(TC4A)_2(V_4O_9)]_3(CyS)_9(PhCOO)_2Cl_3(SO_4)_4(DMF)(H_2O)(CH_3OH)\}^{2+}$	6008.1757	6008.3196

Supplementary Table 6: The operating parameters of ESI-MS of Ag14 and Ag43.

**Operating Parameters**	**Ag14**	**Ag43**
Temperature (°C)	100	100
Capillary voltage (V)	5500	5500
Collision energy (V)	10	0
Quadrapole energy (V)	10	15
Nebulizer (Bar)	0.3	0.3
Nanobooster (Bar)	0.3	0.3
Sample injection rate (μL min^{-1})	20	20
Sample concentration (mol L^{-1})	5×10^{-5}	5×10^{-5}

Supplementary Table 7: Reported solid-state photothermal silver nanomaterials.[7,9,18,19]

Compound	Laser power (W cm^{-2})	Laser wavelength (nm)	Maximum temperature (^{o}C)	Time (min)	Irradiation distance (cm)	Ref.
Ag16(I)	0.1	420-780	81.6	20	/	9
Ag16(II)			72.3			
Ag16(III)			70.4			
Ag16(IV)			70.3			
Ag nanotriangle	10^{5}	740	258.5	/	/	18
Ag nanorod		800	294.3			
Ag NPs@MOF	0.7	808	239.8	0.33	8	19
SD/Ag18a	0.9	660	187	0.33	20	7
**Ag14**	0.9	660	194	0.025	20	This work
**Ag43**			141	0.28		

Supplementary References:

1. Rigaku Oxford Diffraction. *CrysAlis^{Pro} Software system, version 1.171.40.25a*, Rigaku Corporation: Oxford, UK, (2018).
2. Palatinus, L. & Chapuis, G. SUPERFLIP - a computer program for the solution of crystal structures by charge flipping in arbitrary dimensions. *J Appl Crystallogr* **40**, 786-790 (2007).
3. Sheldrick, G.M. Crystal structure refinement with SHELXL. *Acta Crystallogr. C: Struct. Chem.* **71**, 3-8 (2015).
4. Dolomanov, O.V., Bourhis, L.J., Gildea, R.J., Howard, J.A.K. & Puschmann, H. OLEX2: a complete structure solution, refinement and analysis program. *J. Appl. Crystallogr.* **42**, 339-341 (2009).
5. Spek, A.L. Structure validation in chemical crystallography. *Acta. Crystallogr. Sect. D.* **65**, 148-155 (2009).
6. Hoppe, E. & Limberg, C. Oxovanadium(V) tetrathiacalix[4]arene complexes and their activity as oxidation catalysts. *Chem. Eur. J.* **13**, 7006-7016 (2007).
7. Wang, Z. et al. Solvent-controlled condensation of $[Mo_2O_5(PTC4A)_2]^{6-}$ metalloligand in stepwise assembly of hexagonal and rectangular Ag_{18} nanoclusters. *Angew. Chem. Int. Ed.* **61**, e202200823 (2022).
8. Gao, X. et al. Synthesis and near-infrared photothermal conversion of discrete supramolecular topologies featuring half-sandwich [Cp*Rh] units. *J. Am. Chem. Soc.* **143**, 17833-17842 (2021).
9. Wang, H. H. et al. Monocarboxylate-protected two-electron superatomic silver nanoclusters with high photothermal conversion performance. *Nanoscale* **15**, 8245-8254 (2023).
10. Liu, Y. et al. Dopamine-melanin colloidal nanospheres: an efficient near-infrared photothermal therapeutic agent for in vivo cancer therapy. *Adv. Mater.* **25**, 1353-1359 (2013).
11. Hong, S. H., Olin, Å. & Hesse, R. The crystal structure of silver(I) cyclohexanethiolate. *Acta Chem. Scand. A.* **29**, 583-589 (1975).
12. Hong, J. et al. Dimeric water embedded within a hydrophobic cavity of tetra-(*p-tert*-butyl)thiacalix[4]arene. *J. Mol. Struct.* **655**, 435-441, (2003).
13. Voss, N.R. & Gerstein, M. 3V: cavity, channel and cleft volume calculator and extractor. *Nucleic Acids Res* **38**, W555-W562 (2010).

14. Gao, M.-Y. et al. Tetrahedral geometry induction of stable Ag-Ti nanoclusters by flexible trifurcate TiL_3 metalloligand. *J. Am. Chem. Soc.* **142**, 12784-12790 (2020).

15. Wang, Z. et al. Stepwise assembly of Ag_{42} nanocalices based on a Mo^{VI}-anchored thiacalix[4]arene metalloligand. *ACS Nano* **16**, 4500-4507 (2022).

16. Ueda, M., Goo, Z. L., Minami, K., Yoshinari, N. & Konno, T. Structurally precise silver sulfide nanoclusters protected by rhodium(III) octahedra with aminothiolates. *Angew. Chem. Int. Ed.* **58**, 14673-14678 (2019).

17. Yoshinari, N., Goo, Z. L., Nomura, K. & Konno, T. Silver(I) sulfide clusters protected by rhodium(III) metalloligands with 3-aminopropanethiolate. *Inorg. Chem.* **62**, 9291-9294, (2023).

18. Borah, R. & Verbruggen, S. W. Silver-gold bimetallic alloy versus core-shell nanoparticles: implications for plasmonic enhancement and photothermal applications. *J. Phys. Chem. C* **124**, 12081-12094 (2020).

19. Su, J. et al. Enhancing the photothermal conversion of tetrathiafulvalene-based MOFs by redox doping and plasmon resonance. *Chem. Sci.* **13**, 1657-1664 (2022).
